# Leveraging time-series point clouds for dynamic crop canopy monitoring: Quantifying phenotypic variability and assessing leaf-level photosynthetic contributions

**DOI:** 10.1016/j.plaphe.2026.100194

**Published:** 2026-03-04

**Authors:** Jiaren Zhou, Yu Zhang, Man Zhang, Mengqi Zhang, Qingfeng Song, Xin-Guang Zhu, Minjuan Wang

**Affiliations:** a Key Lab of Smart Agriculture Systems, Ministry of Education, College of Information and Electrical Engineering, China Agricultural University, Beijing, 100083, China; b National Key Laboratory of Plant Molecular Genetics, CAS Center for Excellence in Molecular Plant Sciences, Shanghai Institute of Plant Physiology and Ecology, Chinese Academy of Sciences, Shanghai, 200031, China; c Key Laboratory of Agricultural Information Acquisition Technology, Ministry of Agriculture and Rural Affairs, China Agricultural University, Beijing, 100083, China

**Keywords:** Time-series plant phenotyping, Point cloud segmentation, Skeleton extraction, Leaf-level growth tracking, Canopy photosynthesis, Phenotypic variation rate

## Abstract

Time-series point clouds have emerged as an effective approach for precise, continuous crop monitoring and quantitative growth analysis. This study constructed a spatiotime-series point cloud dataset containing four species and eleven plant varieties, exploring crop organ instance segmentation, phenotypic parameter extraction, growth quantification, and canopy photosynthesis assessment. A skeleton-based framework for organ-level instance segmentation and time-series analysis is proposed, demonstrating robust performance across all four crops. To fully utilize the time-series data, a novel time-series leaf matching method was introduced, achieving a matching accuracy, defined as the proportion of correctly matched leaves, of over 0.823 for all species. By integrating the matching results with phenotypic parameter extraction, time-series phenotypic data were generated, and a phenotypic variation rate was defined as a suitable metric for quantifying crop growth. Furthermore, these results were integrated into a canopy photosynthesis model to derive key time-series photosynthetic metrics, including photosynthetic rate, absorbed light quantity, light energy utilization efficiency, and each crop organ's contribution to photosynthesis. These metrics provide insights into the crop's growth patterns and photosynthetic strategy. This study offers refined quantitative analysis of crop morphology and photosynthetic parameters through time-series point cloud segmentation, contributing valuable data for advancing plant biology research and enhancing the understanding of crop growth dynamics.

## Highlights

• Skeleton-guided 3D segmentation and tracking: Developed a robust method for organ-level point cloud segmentation and time-series matching, enabling accurate monitoring of crop development.
• Leaf-scale growth and photosynthesis quantification: Established a pipeline to track leaf-level growth and compute key photosynthetic traits, including photosynthetic rate, absorbed light, and light use efficiency.
• Phenotypic Variation Rate (PVR): Proposed a new metric to quantify dynamic changes in 3D plant structure, enhancing the analysis of growth patterns across time.

## 1 Introduction

Phenotype represents the observable traits of crops that arise from the interaction between genotype and environment, encompassing both morphological and physiological characteristics [1]. Among these, structural traits such as plant height, leaf area, and leaf angle are key indicators of crop growth dynamics and biomass accumulation [2]. Quantitative assessment of these traits is essential for understanding crop development and yield potential. In recent years, high-throughput phenotyping technologies have enabled large-scale and automated measurements of crop morphology. However, many conventional approaches rely on two-dimensional images or spectral data, which are limited by occlusion and projection distortions, making it difficult to capture the true three-dimensional structure of plants over time [3,4]. The challenges mentioned above have driven the advancement of three-dimensional (3D) sensing techniques, such as light detection and ranging (LiDAR) [5] and multi-view stereo imaging, to achieve accurate and non-destructive characterization of crop architecture throughout the growth cycle. Such 3D sensing technologies have found extensive applications in diverse environments, including open-field crop monitoring [6], greenhouse experiments [7], and forestry investigations [8], enabling precise quantification of plant structures under varying conditions. Depending on the spatial scale and target species, 3D sensing systems can be integrated into multiple platforms, such as unmanned aerial vehicles (UAV) [9], ground-based vehicles [10], or stationary setups [11]. Collectively, these multi-platform strategies have greatly broadened the scope of 3D phenotyping, facilitating detailed capture of crop architectural traits across both temporal and spatial scales.

Currently, most research on crop phenotyping focuses on single time points at specific growth stages, such as the seedling stage [12] or the tillering stage [13], etc. While time-series data, with their unique spatiotemporal characteristics, offers a promising approach to overcome these limitations. Continuous phenotypic monitoring and analysis throughout the entire growth cycle, however, remain limited. Most existing time-series phenotyping studies have been conducted at field scales using UAV- [14] or satellite-based [15] remote sensing to monitor crop phenology and large-scale growth dynamics [16], where the temporal intervals between acquisitions are relatively long, typically corresponding to distinct growth stages. Fine-scale, short-interval observations of individual plants (e.g., daily changes) are still rare, limiting the understanding of continuous organ-level growth processes. These limitations mainly stem from the difficulty of continuously acquiring and accurately annotating long-term data, as well as from the challenge of achieving high-precision, point-level organ instance segmentation across multiple species and growth stages. To address these challenges, time-series point cloud data offer a promising solution. By enabling accurate matching of crop organs across consecutive time points, this approach allows the tracking of organ development and senescence, thereby revealing dynamic growth patterns and underlying developmental trends at a finer scale.

To enable organ-level characterization from time-series point clouds, skeleton extraction has become a prevalent approach for capturing the topological and structural attributes of crops. Traditional skeletonization techniques, such as Laplacian-based iterative contraction algorithms, can effectively reduce the complexity of point clouds while preserving the global plant architecture [17]. These methods are generally straightforward, parameterizable, and adaptable to diverse plant point cloud datasets [18]. Nevertheless, several limitations arise when applied to crop structures. One prominent challenge is the emergence of redundant skeleton branches in regions with extensive leaf coverage due to uneven point cloud density, which complicates the delineation of individual organs [19]. Another critical issue is the deviation of stem skeletons from the anticipated linear geometry at leaf-stem junctions, as branching structures can bias skeleton nodes toward adjacent leaves [20]. Furthermore, conventional skeleton graphs typically lack semantic or instance-level information, constraining their applicability for precise organ segmentation and dynamic phenotypic analyses [21]. These limitations highlight the urgent need for skeleton extraction methods capable of accurately capturing organ-level structures with semantic and instance-level information.

The crop canopy, comprising all above-ground organs including both leaf and non-leaf tissues, determines overall photosynthetic performance through the combined efficiency of these organs [22]. Because improving canopy photosynthetic efficiency is a key strategy for enhancing crop yield potential [[23], [24], [25]], earlier studies have employed 3D point cloud-based models to examine how crop architecture influences photosynthesis [[26], [27], [28]]. However, most existing studies have focused on whole-canopy photosynthesis rather than organ-level contributions [29,30]. Incorporating organ-level information into canopy models enables the characterization of diurnal photosynthetic dynamics and quantification of each organ's contribution. Such analyses of organ-specific contribution times and proportions across growth stages can inform breeding strategies aimed at enhancing crop yield.

This study focuses on time-series crop point clouds and proposes a skeleton-based method for point cloud segmentation and organ instance extraction. By aligning leaves across consecutive time points, dynamic morphological traits and quantitative canopy photosynthesis parameters can be derived. This framework enables systematic monitoring of crop growth and photosynthesis at both organ and canopy levels, facilitating quantitative analysis of temporal morphological and photosynthetic dynamics and providing insights into organ-level contributions to canopy photosynthesis.

## 2 Materials and methods

### 2.1 Data acquisition and preprocessing

Both publicly available datasets and self-collected data were used. The self-collected data experiments were conducted in 2022 at the Songjiang experimental station of the CAS Center for Excellence in Molecular Plant Sciences (CEMPS) (latitude 30°94′ N, longitude 121°13′ E). In this study, maize cultivars (W64A, B73, and A619), soybean cultivars (D21020, D21116, and D38), and rice cultivars (M107, M55, and M56) are designated by their assigned identification numbers. The crop varieties and corresponding data collection time points are summarized in Table 1. Due to the late planting of maize variety A619, daily data were collected during its early growth stage (July 29-August 5) and on four selected dates in the middle stage (August 7, 9, 11, 15). The collection spanned 18 days with 12 time points. Other self-collected datasets used continuous time points. Hereafter, this study uses days after planting (DAP) to represent time, with the planting date designated as DAP 1.

**Table 1:** Information on crop varieties and time points.

Species	Variety	Planting Date	Start Date of Shooting	End Date of Shooting	Number of Data Time Points
maize	W64A, B73	7.12	7.19	8.2	15 days
	A619	7.20	7.29	8.15	12 days
soybean	D38, D21020, D21116	7.16	7.24	8.2	10 days
rice	M55, M56, M107	7.13	7.24	7.30	7 days
maize	Pheno4D	-	3.13	3.25	12 days
tomato	Pheno4D	-	3.05	3.25	20 days

Maize and soybean were cultivated in 20 L white plastic buckets, while rice was grown in 7 L black pots, all placed outdoors under natural conditions. The containers were filled with potting soil (garden soil: peat: vermiculite = 5:3:2). For each crop variety, three pre-germinated seeds were planted 30 mm deep in the soil. Three days after germination, thinning was carried out to retain only one uniformly growing plant per pot. Initially, 15 biological replicates were maintained for each variety. During the tillering stage, plants exhibiting poor growth were promptly excluded to ensure the dataset's quality and consistency. To better validate the effectiveness of the algorithm, in addition to the self-collected dataset, this study also used the publicly available point cloud data from the Pheno4D dataset [31], which includes point cloud data from 7 maize plants and 7 tomato plants, totaling 84 maize data samples and 140 tomato data samples.

The data collection was conducted using the 64-camera multi-view stereo system (MVS-64) [32,33]. This system, controlled by a computer, simultaneously captures images from 64 Canon EOS 1300D digital cameras (24-megapixel, Canon Inc., Japan), with approximately 75% overlap between adjacent views. Accurate crop phenotypes (plant height, canopy width, leaf count, leaf length, leaf width, and leaf angle) were manually measured with a ruler and protractor during multi-view image capture and then processed using Agisoft Metashape Professional software (Version 1.7.4, Agisoft LLC, Russia). The raw point cloud contained a significant amount of noise, requiring preprocessing to obtain valid plant point clouds. Due to the significant color difference between the plants and the noise, HSV color thresholding was used to remove the noise. Even after applying color thresholding, many scattered points remained, which were removed using statistical filtering and radius filtering.

Farthest point sampling (FPS) was used for uniform downsampling, reducing the original point cloud to 4096 points. CloudCompare software (Version 2.10, Open Source, France) was used to annotate the point cloud data. For each sequence of point clouds collected from the same plant at different time points, both semantic labels (stem = 1, leaf = 2) and instance labels (stem instance = 1, leaves numbered sequentially starting from 2) were assigned. Within the entire time-series sequence of a single plant, a consistent instance label was maintained for each leaf. During annotation, the leaf-stem junctions were labeled using a top-down perspective. Polygonal segmentation tools were used to trace the junction line. Multiple segmentation steps were performed to separate individual leaf instances, and segmented leaf fragments were merged to form complete leaf instances.

### 2.2 Crop skeleton extraction and optimization pipeline

A crop skeleton representation was generated to enable organ-level instance segmentation and time-series analysis. Point cloud skeletons are widely used to capture the intrinsic topological and structural characteristics of crops, transforming irregular 3D point distributions into compact representations suitable for structural analysis. The Laplacian-based iterative contraction algorithm [34] was employed in this study owing to its efficiency, flexibility, and capability to preserve global topology while simplifying dense point clouds. Specifically, the algorithm takes the plant point cloud dataset $P\in R^{N_{P}\times 3}$ as input and outputs a zero-volume contracted set C, where $N_{P}$ denotes the number of points. Farthest point sampling is subsequently applied to downsample C and obtain skeleton points U, which are then connected through edge contraction operations (contraction control parameter α = 0.008) to form the edge set E. The resulting undirected skeleton graph G=(U,E) provides a compact representation of the plant architecture.

The iterative contraction framework can introduce artifacts in regions of dense leaf coverage and near branching junctions, such as redundant branches or stem deviations. The skeleton graph also lacks semantic and instance-level information. To address these issues, an improved laplacian skeleton extraction method was implemented, including topological node classification, redundant branch pruning, and stem calibration. The optimization process begins by classifying skeleton nodes according to their topological connectivity. Let d(u) denote the degree (number of incident edges) of node u∈U in the graph G. Nodes with d(u)≥3 are recognized as stem nodes, those with d(u)=2 as connection nodes, and those with d(u)=1 as leaf tip nodes (Fig. S1). An exception is made for the skeleton node located at the plant base, which, despite being connected by a single edge, is identified as a stem node because of its minimal *z*-coordinate and functional role as the plant's structural origin. Here the z-coordinate is defined as the vertical axis in metres; the node with the minimum z among degree-1 nodes is considered the stem base.

Based on the identified key nodes, redundant branches were systematically removed. For each leaf tip node ${\text{leaf}}_{i}\in U$, the shortest path ${\text{road}}_{i}$ between ${\text{leaf}}_{i}$ and the stem base node stem_node was computed. When two paths ${\text{road}}_{i}$ and ${\text{road}}_{j}$ overlapped by more than 75% of their total length and had negligible length difference, the shorter path was removed. The skeleton graph was then reconstructed using the preserved paths. To recalibrate the stem skeleton, a three-dimensional linear fitting based on singular value decomposition (SVD) was applied. After computing the centroid $C\left(x_{0},y_{0},z_{0}\right)$ of the stem point cloud and centralizing the coordinates, the eigenvector corresponding to the largest eigenvalue is extracted as the stem direction vector K=(a,b,c). Here, C is the arithmetic mean of the 3D coordinates of the stem point, and K is a unitless direction vector whose components correspond to direction cosines along the x, y and z axes. The fitted stem line is described by Eqn (1):

Eqn 1 $$\begin{array}{c} \frac{x-x_{0}}{a}=\frac{y-y_{0}}{b}=\frac{z-z_{0}}{c} \end{array}$$

Here, the triplet (x,y,z) denotes a generic point along the fitted stem line in metres; $\left(x_{0},y_{0},z_{0}\right)$ is the centroid; and (a,b,c) are the components of K. Each stem node $\left(x_{1},y_{1},z_{1}\right)$ is then projected onto this line according to Eqn (2):

Eqn 2 $$\begin{array}{c} \left\{ \begin{array}{c} \begin{array}{l} k=\frac{\left|\left(x_{1}-x_{0}\right)a+\left(y_{1}-y_{0}\right)b+\left(z_{1}-z_{0}\right)c\right|}{a^{2}+b^{2}+c^{2}}\\ x^{\prime }=x_{0}+k\cdot a \end{array}\\ \begin{array}{l} y^{\prime }=y_{0}+k\cdot b\\ z^{\prime }=z_{0}+k\cdot c \end{array} \end{array}\right. \end{array}$$

Here, the scalar k is the signed scalar projection of the vector $\left(x_{1}-x_{0},y_{1}-y_{0},z_{1}-z_{0}\right)$ onto the direction vector K; $\left(x^{\prime },y^{\prime },z^{\prime }\right)$ are the coordinates of the orthogonal projection of the original node onto the fitted line. This procedure aligns the stem skeleton with the vertical axis. All mathematical symbols, variables, and parameters used in the algorithm are defined in Table 2, and the workflow of the pipeline is summarized in Fig. S11.

**Table 2:** Definitions of mathematical symbols and parameters.

Symbol	Definition/Physical Meaning	Unit
P	Original plant point cloud dataset, represented as an $N_{P}\times 3$ matrix where each row is a 3D coordinate (x,y,z).	m
$N_{P}$	Total number of points in the plant point cloud.	-
C	Contracted point cloud obtained through Laplacian-based iterative contraction.	m
U	Set of skeleton nodes obtained after farthest point sampling.	m
E	Set of skeleton edges connecting nodes in U	m
G=(U,E)	Undirected skeleton graph representing plant topology.	-
α	Contraction control parameter that determines the contraction strength or step size.	-
d(u)	Degree of node u, the number of edges incident to node u.	-
${leaf}_{i}$	$i_{th}$ leaf tip node (degree = 1).	-
stem_node	Skeleton node representing the stem base (minimum z-coordinate among degree-1 nodes).	-
${road}_{i}$	Shortest path between ${\text{leaf}}_{i}$ and stem_node within G.	m
$C\left(x_{0},y_{0},z_{0}\right)$	Centroid of the stem point cloud, representing the geometric center.	m
K(a,b,c)	Direction vector of the stem axis obtained via SVD; represents direction cosines along x, y, z axes.	-
(x,y,z)	Generic point coordinate in 3D Cartesian space.	m
$\left(x_{1},y_{1},z_{1}\right)$	Original coordinates of a stem skeleton node before projection.	m
$\left(x^{\prime },y^{\prime },z^{\prime }\right)$	Projected coordinates of a stem skeleton node after calibration.	m
k	Scalar projection of the vector from the centroid to the node onto the stem direction vector K; represents distance along the stem axis.	m

### 2.3 Instance segmentation and phenotypic parameter extraction of crop point clouds

**Fig. 1:**
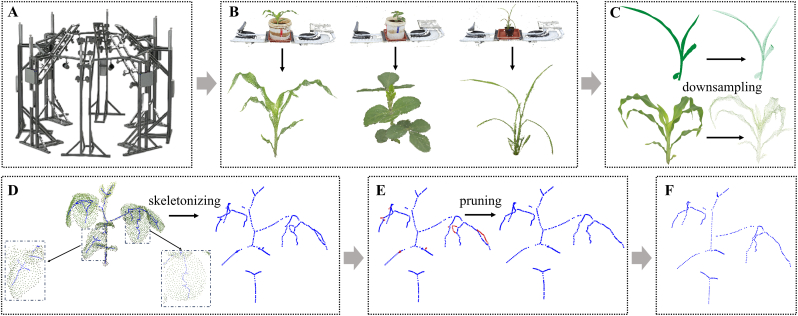
Instance segmentation and phenotypic analysis workflow of crop point clouds. (A) Leaf-level segmentation of crop point clouds. After segmenting the skeleton of the crop point cloud, instances are coarsely segmented based on a distance threshold. For black points that were not successfully segmented, their instances are recalculated to achieve fine segmentation. (B) Canopy-level phenotype extraction. The calculation principles of plant height and canopy width are presented. (C) Leaf-level phenotype extraction. Leaf length is defined as the shortest path along the leaf skeleton between the leaf base point and the leaf tip point. Leaf width is calculated using two cutting planes (S_1_ and S_2_) constructed at a skeleton point based on the normal vectors $\underset{v_{1}}{\to }$ and $\underset{v_{2}}{\to }$ toward its two adjacent skeleton points; the maximum Euclidean distance between points within the cutting planes represents the leaf width. To improve measurement accuracy, the cutting planes are shifted upward by 5 mm along the normal direction. The leaf angle α is defined as the angle ∠MON, where M is a point on the z-axis and N is a point located at one-fourth of the leaf skeleton path.

Based on the optimized skeleton representation, organ-level instance segmentation was performed on the crop point cloud skeleton. Each point in the original point cloud was assigned to the closest skeleton point to determine its organ membership. Initially, coarse segmentation was conducted using a distance threshold. A KD tree was constructed to query all points within the threshold distance from the skeleton anchor points. The threshold distance was defined as the average Euclidean distance between an anchor point and its two neighboring skeleton points. After coarse segmentation, some points remained unclassified (Fig. 1A), and fine segmentation was performed by calculating the Euclidean distance between each unclassified point $P_{i}$ and all skeleton points, assigning it to the organ corresponding to the minimum distance.

Four evaluation indicators were used to assess organ instance segmentation: precision, recall, F1-score, and accuracy. For each organ, true positives ($TP_{i}$), false positives ($FP_{i}$), and false negatives ($FN_{i}$) were counted, and precision $P_{i}$ and recall $R_{i}$ were calculated using Eqn (3) and Eqn (4). Crop-level precision, recall, F1-score, and accuracy were calculated using Eqn (5)–(8).

Eqn 3 $$\begin{array}{c} P_{i}=\frac{{TP}_{i}}{{TP}_{i}+{FP}_{i}} \end{array}$$

Eqn 4 $$\begin{array}{c} R_{i}=\frac{{TP}_{i}}{{TP}_{i}+{FN}_{i}} \end{array}$$

Eqn 5 $$\begin{array}{c} Precision=\frac{\sum_{i=1}^{N}P_{i}}{N} \end{array}$$

Eqn 6 $$\begin{array}{c} Recall=\frac{\sum_{i=1}^{N}R_{i}}{N}\; \end{array}$$

Eqn 7 $$\begin{array}{c} F1-score=2\times \frac{Precision\times Recall}{Precision+Recall} \end{array}$$

Eqn 8 $$\begin{array}{c} Accuracy=\frac{\sum_{i=1}^{N}{TP}_{i}}{n} \end{array}$$

Phenotypic parameters were extracted from the segmented organ point clouds. Plant height was calculated as the difference between the maximum and minimum z-coordinates. Canopy width was determined by projecting the point cloud onto the xOy plane, computing the convex hull using the graham scan algorithm (GSA), and measuring the euclidean distance between the farthest pair of convex hull points (Fig. 1B). Leaf-level parameters, including length, width, and angle, were computed as follows. Leaf length was defined as the shortest path between the base and tip points of the leaf skeleton. Leaf width was determined by constructing cutting planes along the skeleton and measuring the maximum distance between points intersecting the planes (Fig. 1C). Leaf angle was calculated as the angle between vectors defined from the leaf base to points on the skeleton (Eqn (9)).

Eqn 9 $$\begin{array}{c} \alpha =\arccos\left(\frac{\to OM\cdot \to ON}{\left|\to OM\cdot \to ON\right|}\right) \end{array}$$

### 2.4 Time-series point cloud matching of crop organ

A skeleton-guided organ-level matching strategy was implemented to establish consistent instance labels for crop organ point clouds across time series, using crop skeletons as the reference for matching organ instances at adjacent time points. The procedure consisted of the following steps:

(1) Point cloud segmentation. The crop point cloud skeleton point sets $P_{t}$ and $P_{t+1}$ with instance labels at two adjacent time points were segmented into $N_{t}$ and $N_{t+1}$ organ sets according to the instance label information.
(2) Organ count comparison. The numbers of organs $N_{t}$ and $N_{t+1}$ were compared. When $N_{t}>\;N_{t+1}$, the difference was recorded as potential leaf senescence or detachment; when $N_{t}<\;N_{t+1}$, the difference was recorded as potential new leaf emergence.
(3) Distance matrix construction. The Euclidean distances between the centroids of each organ at time t and each organ at time t+1 were calculated to construct a distance matrix M.
(4) Organ matching. The minimum element ${organ\_match\_distance}_{i,j}$ in matrix M was selected as the matching distance for a pair of organs, assigning the *i*th organ at time t corresponded to the j th organ at time t+1. When more than one organ at time t was matched to the same organ at time t+1, only the pair with the smallest matching distance was retained; the remaining organs were matched again using the updated distance matrix.
(5) Leaf status assessment. When $N_{t}\neq N_{t+1}$, the mean matching distance of all matched leaves in the plant was computed. For each unmatched or ambiguously matched leaf, its matching distance and centroid z-value were compared with these reference values. Leaves with a matching distance greater than the mean and a centroid z-value higher than the whole-plant centroid were labeled as newly emerged leaves, whereas leaves with a matching distance greater than the mean and a centroid z-value lower than the whole-plant centroid were labeled as senescent or detached leaves.

Using the above methods, changes in crop organs between adjacent time points were tracked, including leaf emergence and senescence. Three indicators were constructed to quantify the results of time-series point cloud matching:

(1) Matching accuracy (MA): the proportion of organs $N_{T}$ t that are correctly matched to the total number of organs $N_{s}$, calculated by Eqn (10).
  Eqn 10 $$\begin{array}{c} MA=\frac{N_{T}}{N_{S}} \end{array}$$
(2) Matching accuracy of new leaves (MANL): the proportion of newly emerged leaves $N_{new}$ that are correctly matched to the total number of new leaves $N_{sum\_new}$, calculated by Eqn (11).
  Eqn 11 $$\begin{array}{c} MANL=\frac{N_{new}}{N_{sum\_new}} \end{array}$$
(3) Matching accuracy of withered leaves (MAWL): the proportion of senescent or fallen leaves $N_{fallen}$ that are correctly matched to the total number of senescent leaves $N_{sum\_fallen}$, calculated by Eqn (12).
  Eqn 12 $$\begin{array}{c} MAWL=\frac{N_{fallen}}{N_{sum\_fallen}} \end{array}$$

A “correct match” was defined as a leaf instance corresponding to the same physical organ at two adjacent time points, based on spatial overlap and continuity of morphological attributes. MA measures overall temporal consistency, while MANL and MAWL measure the matching of newly emerged and senescent leaves, respectively. These indicators account for continuous organ emergence and senescence in the time-series point clouds.

### 2.5 Description of dynamic crop growth variability

Based on organ-level point cloud matching across time points, phenotypic measurements from individual growth stages were integrated into time-series datasets. Dynamic growth variability at both the whole-plant and organ levels was calculated using the phenotypic variation rate (PVR). For day t, PVR is defined as Eqn (13).

Eqn 13 $$\begin{array}{c} {PVR}_{t}=\frac{P_{t}-P_{t-1}}{P_{t-1}}\times 100\%\;\left(t\geq 2\right) \end{array}$$

Where $P_{t}$ is the phenotypic value on day t. PVR is calculated as a direct percentage change, in contrast to the log-based normalization of the relative growth rate (RGR) [35]. The average phenotypic variation rate over a period of D days, denoted $\bar{{PVR}_{D}}$, is calculated as Eqn (14).

Eqn 14 $$\begin{array}{c} \bar{{PVR}_{D}}=\frac{100\%}{D-1}\times \sum_{t=2}^{D}{PVR}_{t}\;\left(t\geq 2\right) \end{array}$$

Daily and average PVR values were computed for each phenotypic trait in the time-series dataset.

### 2.6 Simulation of crop canopy photosynthesis

To construct a realistic model of the crop canopy, a single plant sample was used and was replicated, randomly rotated around the z-axis, and translated according to predefined spatial arrangements. Specifically, the point cloud of this sample was duplicated 16 times. Each replicated plant was randomly rotated by a certain angle around the vertical axis and positioned with a plant spacing of 200 mm and a row spacing of 500 mm, resulting in a three-dimensional maize canopy structure with 16 plants arranged in 4 rows × 4 columns (Fig. S2).

Using the ray tracing software FastTracer software [36,37], light distribution was simulated and the photosynthetic photon flux density (PPFD) was calculated for each leaf surface patch. In the simulation, the model space size and latitude (30°N) were specified, with atmospheric transmittance set to 0.7 (corresponding to clear-sky conditions), leaf transmittance set to 0.2, leaf reflectance set to 0.25, and solar azimuth and elevation angles set to 45°. Meteorological data from days 246 to 286 of the year were incorporated, including temperature, relative humidity (RH), total PPFD, and diffuse PPFD, obtained from historical records of the local weather station. For each leaf surface patch represented as a triangular mesh, contributions from direct sunlight, atmospheric scattering, and reflected light from other surfaces were computed on both the front and back sides, allowing calculation of the light environment of individual leaf surfaces (Fig. S12).

These PPFD values were then input into a non-rectangular hyperbola model fitted to the leaf photosynthetic light response (A-Q) curves, which were measured using the LI-6400XT Portable Photosynthesis System (LI-COR Biosciences, USA) under light intensities of 0-2000 μmol m^−2^ s^−1^ (6:00-18:00, hourly). Measurements were conducted on each individual plant, selecting the middle-upper leaves (third leaf from the top) for maize and soybean, and the flag leaf for rice. Simulations ran from 6:00 to 18:00 at 1-h intervals, under light quantum flux densities ranging from 0 to 2000 μmol m^−2^ s^−1^, with leaf temperature fixed at 25 °C. A-Q curve parameters were set as follows: quantum yield (φ) of 0.1, maximum photosynthetic rate ($P_{\max}$) of 30, dark respiration rate ($R_{d}$) of 1, and curvature factor (θ) of 0.5.

The canopy photosynthetic parameters were calculated using the simulation outputs and the instance label information of each leaf, including total absorbed light (Eqn (15)), Light intensity (LI) (Eqn (16)), light energy utilization efficiency (LUE) (Eqn (17)), and photosynthetic rate. The canopy photosynthetic rate is calculated as the total CO_2_ absorption for photosynthesis divided by the ground area. The daily net canopy photosynthetic CO_2_ absorption rate per unit ground area, $A_{c}$, is shown in Eqn (18), where $S_{i}$ represents the area of the ith leaf segment, ${IF}_{i}$ represents the PPFD value of the ith leaf, and A(0) represents the photosynthetic rate of the leaf under zero incident light. The ground area, $S_{g}$, is the area of the central region of the four plants. This study investigates the impact of different plant types on the canopy photosynthetic rate.

Eqn 15 $$\begin{array}{c} light_{absorbed}=\sum_{i=1}^{n}S_{i}\times {IF}_{i} \end{array}$$

Eqn 16 $$\begin{array}{c} LI=\frac{\int_{t=6}^{18}\left(\sum_{i=1}^{n}S_{i}\times {IF}_{i}\right)dt}{S_{g}}\; \end{array}$$

Eqn 17 $$\begin{array}{c} A_{c}=\frac{\int_{t=6}^{18}\left(\sum_{i=1}^{n}S_{i}\times A\left({IF}_{i}\right)\right)dt-12\times 3600\times \sum_{i=1}^{n}S_{i}\times A\left(0\right)}{S_{g}}\; \end{array}$$

Eqn 18 $$\begin{array}{c} LUE=\frac{A_{c}}{LI}\; \end{array}$$

## 3 Results

### 3.1 Crop instance segmentation and phenotype extraction

To evaluate instance segmentation accuracy, 50 samples were selected for each crop type. For each variety, one sample included all time points of a single plant to ensure coverage of the time-series dimension, while the remaining samples were randomly selected across all time points to ensure diversity and representativeness. The segmentation accuracy results are presented in Table 3. Taking maize as an example, compared with existing non-deep-learning point cloud segmentation algorithms, such as the maize stem and leaf segmentation method based on skeleton extraction using optimal transport distance (accuracy = 0.967) [38], the skeleton extraction and correction-based approach used in this study achieved a 0.7% improvement in accuracy, while requiring no significant increase in computational resources. In terms of testing on public Pheno4D dataset, the instance segmentation accuracy for corn and tomato improved by 0.174 and 0.109 percentage points, respectively, compared to the baseline.

**Table 3:** Instance segmentation accuracy based on skeleton extraction and calibration.

Precision	Recall	F1-scores	Accuracy	Crop Species
0.973	0.965	0.969	0.974	self-collected dataset maize
0.980	0.974	0.977	0.982	Pheno4D dataset maize
0.879	0.872	0.875	0.906	self-collected dataset soybean
0.851	0.829	0.840	0.805	Pheno4D dataset tomato
0.751	0.745	0.748	0.774	self-collected dataset rice

**Fig. 2:**
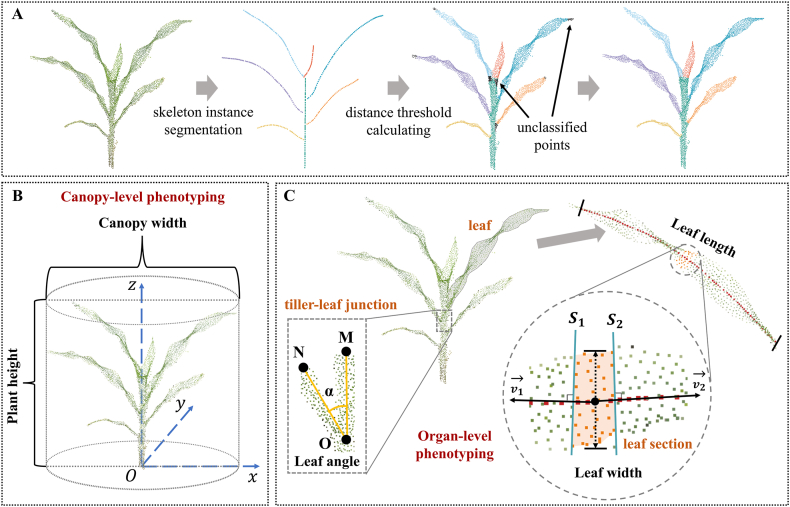
Crop point cloud instance segmentation results, arranged in chronological order according to days after planting (DAP). (A) Instance segmentation results for self-collected maize, The blue circled area indicates an incorrect segmentation due to leaf interruption. (B) Instance segmentation results for self-collected soybean, The blue circled area indicates an incorrect segmentation because the soybean leaves are relatively wide, making it easy to confuse the leaf base with the stem tiller region. (C) Instance segmentation results for self-collected rice, The blue circled area indicates an incorrect segmentation as the leaf bases of different leaves are also prone to confusion. (D) Instance segmentation results for maize and tomato in Pheno4D dataset. The supplementary information of all data are shown in Fig.S3, Fig.S4, Fig.S5 and Fig.S6.

To visually demonstrate the effects of crop point cloud instance segmentation, representative time points from different species were selected for visualization, presented in Fig. 2. At the same time points, different colors were used to represent different segmentation instances to clearly display the segmentation effects. However, it should be noted that even if the colors are the same at different time points, it does not indicate that they have the same instance label. Complete instance segmentation effect images can be found in Fig. S3, Fig. S4, Fig. S5 and Fig. S6.

Based on the data in Table 2 and the segmentation results in Fig. 2, it is evident that the proposed point cloud instance segmentation method based on skeleton extraction has high accuracy and demonstrates good performance across different crops. This advantage is primarily attributed to the fine optimization of the extracted skeletons, which effectively removed redundant skeletons, resulting in a more complete overall morphology of the segmented crop organs. Furthermore, this method effectively avoids the missegmentation of the same organ into different instances, further enhancing the accuracy and reliability of instance segmentation.

For the structurally simpler monocot crop, maize, both self-collected and public datasets achieved high segmentation accuracy, exceeding 0.974, with slightly lower precision observed in the self-collected dataset (Fig. 2A). Skeleton-based segmentation identified individual maize leaves, with occasional missegmentation when adjacent organs were closely spaced or when incomplete leaf point clouds occurred. For dicot crops (soybean and tomato), clear morphological distinctions between leaves and stems and denser leaf point clouds enabled effective segmentation, with occasional errors for partially unfolded or heavily overlapping leaves (Fig. 2B). For rice, a structurally more complex crop, the method remained effective but showed limitations due to the fine and closely spaced leaves, with some missegmentation at leaf bases due to closely spaced leaves (Fig. 2C).

**Fig. 3:**
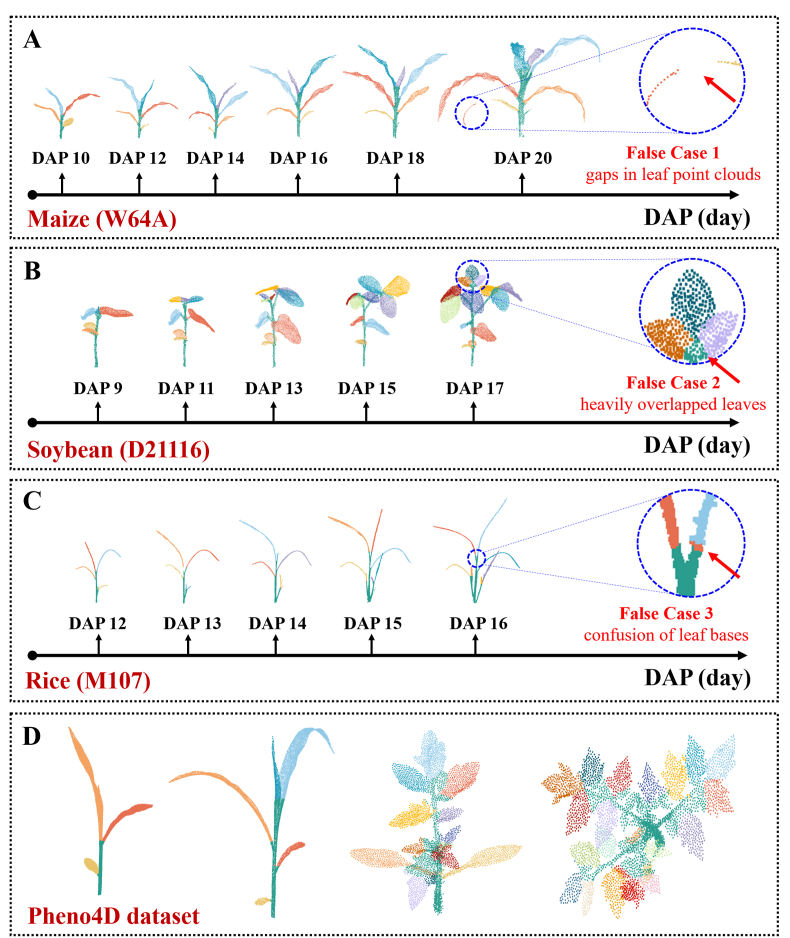
(A) Phenotypic data distribution of three species. (B) Significance analysis of phenotypic data among the three species, conducted using analysis of variance (ANOVA) at a significance level of p = 0.05.

Building on these segmentation results, the proposed framework further evaluated the accuracy of downstream phenotypic measurements across all three crops. For all three crops, extracted phenotypes exhibited strong correlation with measured values (R^2^ > 0.9). Errors were observed in plant height, canopy width, leaf count, leaf length, leaf width, and leaf angle, with variable accuracy across traits. R^2^ values for leaf count and leaf width in rice were below 0.9. Fig. 3A summarizes the statistical analysis of six phenotypic traits across the three crop species, and Fig. 3B presents pairwise significance tests. Most traits showed significant interspecific differences, whereas plant height, leaf number, leaf width, and leaf angle were not significantly different.

### 3.2 Evaluation of leaf-level matching in time series point clouds

**Fig. 4:**
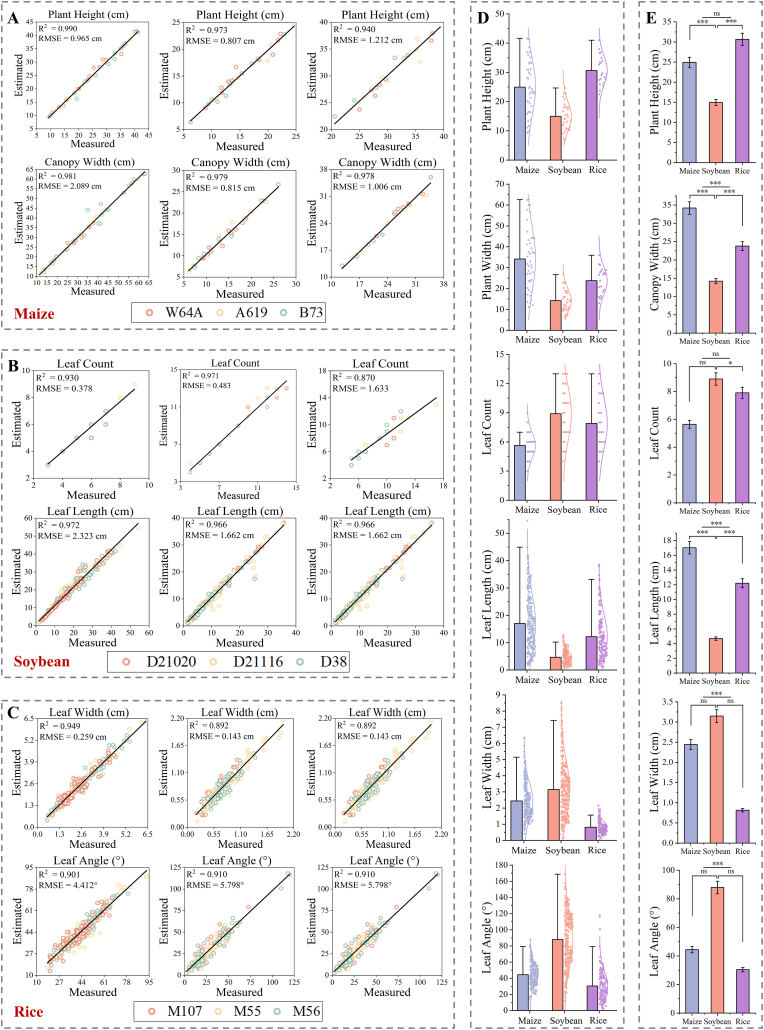
Organ matching results, red circles are mismatched results, arranged in chronological order according to days after planting (DAP). (A) Self-collected dataset maize. (B) Self-collected dataset soybean. (C) Self-collected dataset rice. (D) Many-to-one incomplete matching. (E) Transient temporal-chain break.

Three complete time-series crops were selected for each crop for organ matching, and one of them was selected for visualization. Table 4 shows the organ matching accuracy, and Fig. 4 shows some organ matching results. Since the public dataset does not include leaf shedding, the leaf shedding evaluation index MAWL of the Pheno4D dataset is 1.

**Table 4:** Organ matching accuracy.

	Self-collected dataset maize	Self-collected dataset soybean	Self-collected dataset rice	Pheno4D dataset maize	Pheno4D dataset tomato
MA	0.964	0.869	0.829	0.981	0.823
MANL	0.967	0.889	0.826	0.983	0.833
MAWL	0.970	0.944	0.889	1.000	1.000

The time-series matching algorithm proposed in this study performs well on both self-collected and public datasets, providing reliable support for crop growth research. Maize, with its simple structure and wide spacing between leaves, yields the best matching results, enabling effective identification of new and dropped leaves. Rice, another monocot, performs well in early stages, but accuracy declines as the number of thinner leaves increases. For more complex dicot crops like soybean and tomato, the close proximity of compound leaves leads to significant recognition errors, particularly as overlapping increases in later growth stages. Matching errors can be attributed to two main factors. First, the matching algorithm preserves the closest pairs under a many-to-one configuration and then re-matches the remaining leaves, which can result in many-to-one incomplete matching, as shown in Fig. 4D. Second, growth-induced changes may cause the centroids of two leaves to become temporarily indistinguishable, leading to a transient temporal-chain break, as illustrated in Fig. 4E.

### 3.3 Time-series analysis of dynamic crop canopy phenotypes

**Fig. 5:**
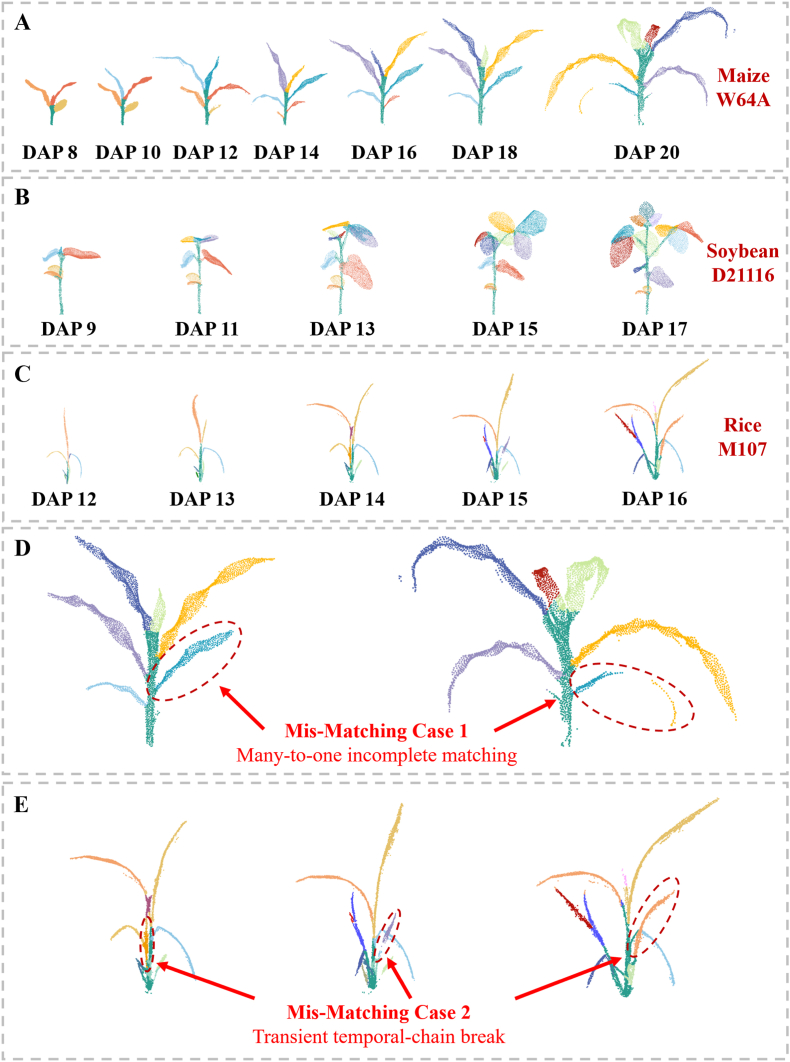
Time-series changes in key phenotypes of maize, soybean, and rice during the seedling stage, arranged in chronological order according to days after planting (DAP). (A) Comparison of temporal growth changes among three species, with indicators including plant height, canopy width, leaf Count, mean leaf length, mean leaf width, mean leaf angle. (B) Time-series variation in leaf length, leaf width and leaf angle of individual maize leaves.

Fig. 5 and Fig. S7 illustrate the time-series changes in crop phenotypes during the seedling stage. Temporal variations in plant height, canopy width, leaf count, and average leaf length, width, and angle were examined across DAP for maize, soybean, and rice Temporal variations in plant height, canopy width, leaf count, and average leaf length, width, and angle were recorded across DAP for maize, soybean, and rice (Fig. 5A). At the same DAP, maize and rice had similar plant height and canopy width, both greater than soybean. In soybean and rice, plant height exceeded canopy width, whereas maize showed slightly larger canopy width than height. Soybean had the highest leaf count, followed by rice, with maize displaying a relatively stable leaf number over time. Average leaf length and width were recorded, with maize having the longest leaves and soybean the widest. Rice showed relatively stable leaf dimensions. Leaf angles were measured for all species, showing variation over time. Maize leaf length, width, and angle varied across DAP, with red circles indicating newly emerged leaves and black squares marking senescence or abscission (Fig. 5B). Leaf length initially increased and then decreased over the seedling stage, while leaf width and angle fluctuated over time.

**Fig. 6:**
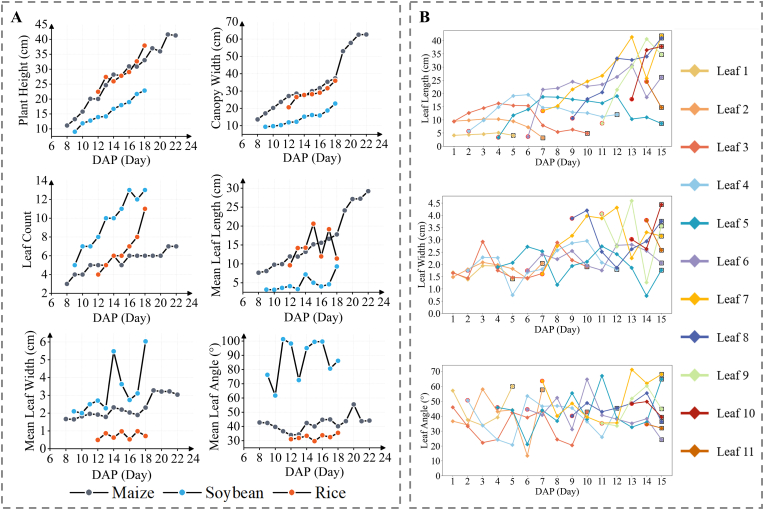
Time-series phenotypic variation rates (PVRs) of the three crops, arranged in chronological order according to days after planting (DAP). (A) PVR variation of maize with DAP. (B) PVR variation of soybean with DAP. (C) PVR variation of rice with DAP.

**Fig. 7:**
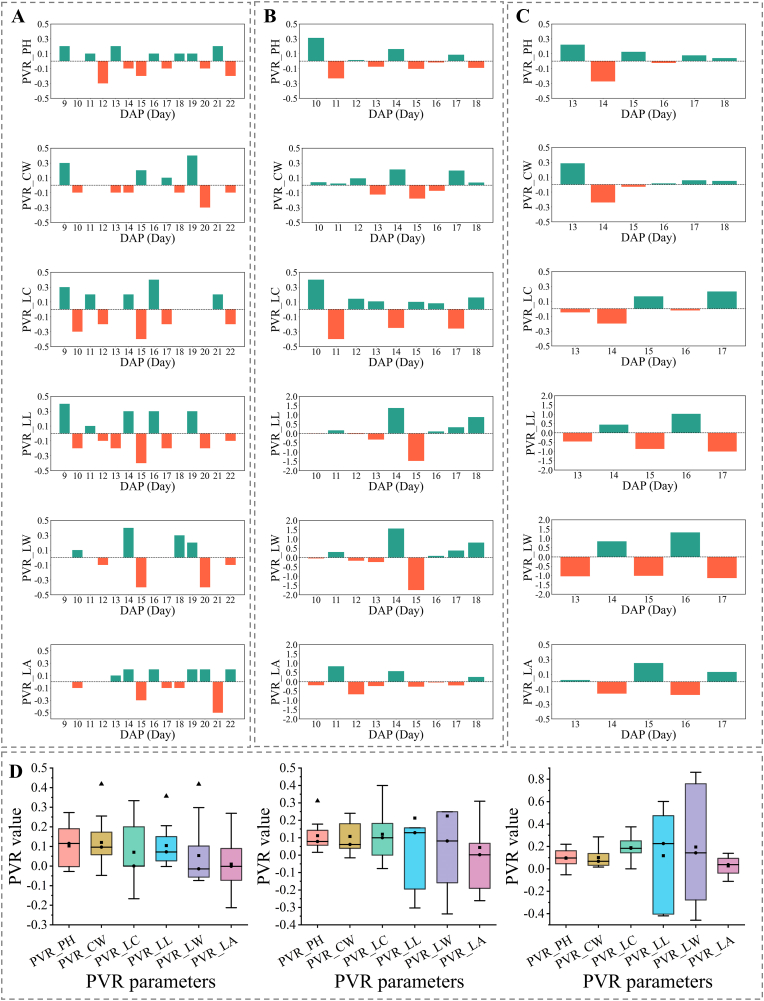
Box plots of PVR values for plant height, canopy width, leaf count, average leaf length, average leaf width, and average leaf angle of maize, soybean, and rice—are denoted as PVR_PH, PVR_CW, PVR_LC, PVR_LL, PVR_LW, and PVR_LA, respectively. The mean values are represented by circular markers, the median values by rectangular markers, and the outliers by triangular markers.

The time-series variation of PVR was examined for maize, soybean, and rice (Fig. 6). In maize seedlings, PVR values varied over time for plant height, leaf length, leaf width, leaf count, and leaf angle. Leaf count exhibited spikes on DAP 9 and 14. Box plot analysis (Fig. 7) summarized PVR distributions for plant height, canopy width, leaf count, leaf length, width, and angle. Species-specific distributions were observed: rice had concentrated PVR distributions for height, canopy width, leaf count, and angle, whereas maize exhibited more stable PVRs for leaf length and width. The PVR of individual maize leaves was examined (Fig. S8). Leaf length and width showed higher variation on the day of emergence, followed by stabilization. Leaf angles were recorded over time. Average PVRs by leaf were calculated (Fig. S9), and interspecific comparisons were performed (Fig. S10).

### 3.4 Time-series quantification of photosynthesis at canopy and leaf levels

**Fig. 8:**
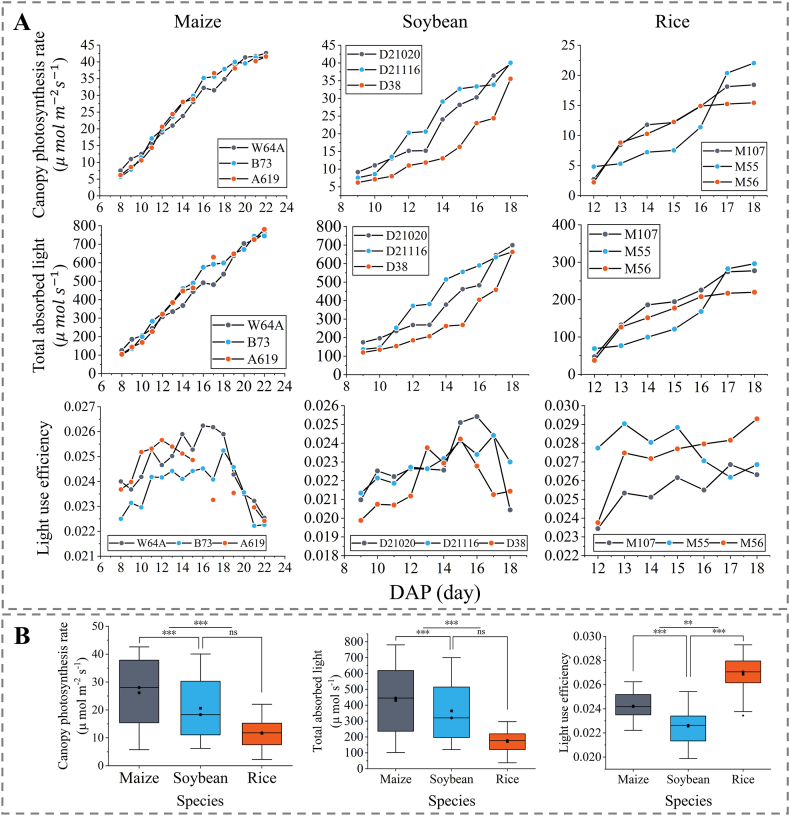
Dynamic canopy photosynthesis quantification of individual crops, arranged in chronological order according to days after planting (DAP). (A) Comparison of temporal photosynthesis changes among three species, with indicators including canopy photosynthesis rate, total absorbed light, light use efficiency (LUE). The values represent the mean of five measurements taken at different time periods throughout the day. The data for DAP 16, 18, and 20 of the maize variety A619 is missing, while the rest are complete and recorded continuously. (B) Box plots illustrate the photosynthesis rate, total absorbed light, and light use efficiency of maize, soybean, and rice, while ANOVA (p = 0.05) was used to analyze the significance of differences among the three species.

Time-series canopy photosynthesis for individual crops was analyzed based on simulations under constant environmental conditions (Fig. 8A). Daily photosynthesis, total absorbed light, and light-use efficiency were recorded over the observation period. Values generally increased as plants developed more leaves. By comparing the differences in photosynthetic parameters among species, a significance analysis was conducted, as shown in Fig. 8B. The canopy photosynthesis rate and total absorbed light of the C4 crop, maize, are significantly different from those of the C3 crops, soybean and rice, while no statistically significant difference is observed between soybean and rice. For LUE, there are marked differences between each pair of species; numerically, rice exhibits the highest LUE, followed by maize, with soybean having the lowest.

**Fig. 9:**
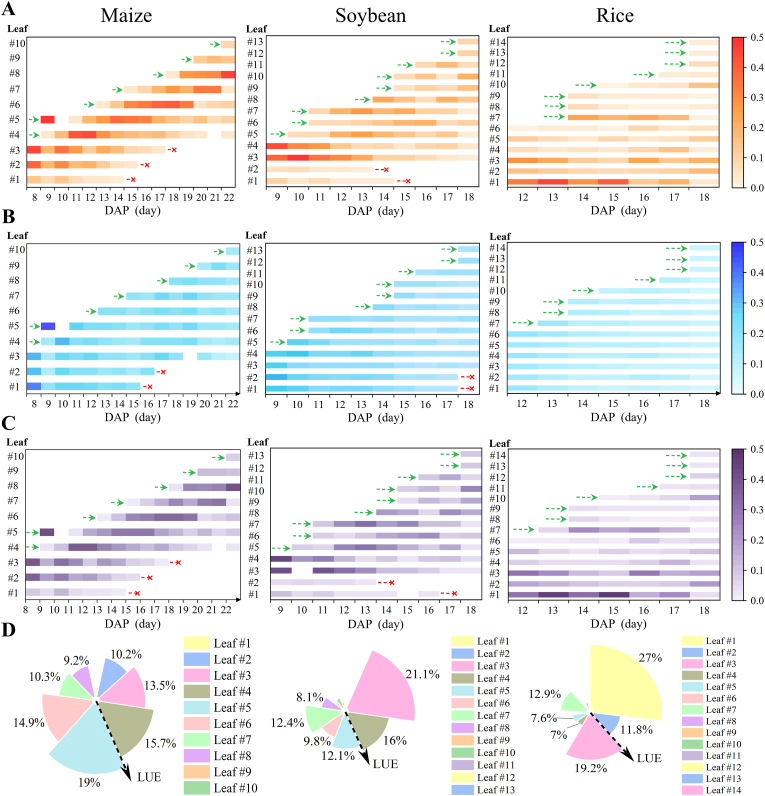
Photosynthetic contribution map of maize, soybean and rice leaves after normalization. Photosynthesis rate, light absorption and light energy utilization efficiency (LUE) are included and represented by color, where darker hues indicate higher data values. The green arrows in the figure indicate newly emerged leaves, while the red “ × ” marks represent senescent and withered leaves, arranged in chronological order according to days after planting (DAP). (A) The photosynthesis rate of each leaf varies with DAP. (B) The light absorption of each leaf changes with DAP. (C) The LUE of each leaf fluctuates with DAP. (D) The total contribution of each leaf's photosynthesis rate over the experimental period is represented by a sector, where the arc length indicates the contribution of the photosynthesis rate, and the sector radius represents the contribution of LUE.

Taking the maize (W64A), soybean (D21116), rice (M107) plant, the results of the canopy photosynthesis quantification analysis at the leaf-level are illustrated in Fig. 9. For example, in maize, from the perspective of photosynthetic rate contribution, 2 to 3 leaves exhibit significantly higher contributions to photosynthetic rates compared to other leaves on a daily basis (functional leaves). In terms of light absorption contribution, the daily light absorption contribution of each leaf becomes more evenly distributed as the plant matures. Regarding light energy use efficiency, individual leaves' contributions to light energy efficiency initially increase and then decrease as the plant grows. In comparison, the time-series photosynthetic contributions of soybean and rice differ significantly from those of maize, with each species exhibiting distinct patterns of photosynthetic contribution changes.

Leaf-level photosynthetic contributions were visualized using a pie chart in which each leaf corresponds to a sector, with the angle representing photosynthetic rate contribution and the radius indicating light-use efficiency (Fig. 9D). Over time, contributions from newly emerged leaves initially increased and then decreased. Leaf 5 had the highest contributions, whereas leaves 1 and 2 contributed less due to early senescence, and leaves 9 and 10 contributed less as they were recently emerged.

### 3.5 Internal correlations of canopy phenotypes and phenotypic variation rates in different species

**Fig. 10:**
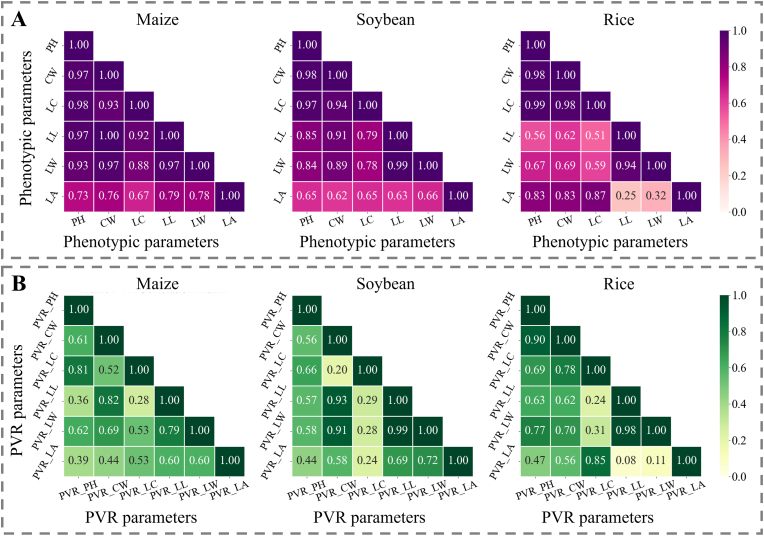
Correlation matrix, from left to right: Maize, Soybean, Rice. Plant height, canopy width, leaf count, average leaf length, average leaf width, and average leaf angle are denoted as PH, CW, LC, LL, LW, and LA, respectively. The phenotypic variation rate (PVR) of plant height, canopy width, leaf count, average leaf length, average leaf width, and average leaf angle are denoted as PVR_PH, PVR_CW, PVR_LC, PVR_LL, PVR_LW, and PVR_LA, respectively. (A) Phenotypic correlation matrix. (B) PVR correlation matrix.

Phenotype correlation matrices were analyzed for each species (Fig. 10A). In maize, plant height, canopy width, leaf count, and average leaf length and width were strongly correlated, whereas leaf angle showed little association with other traits. In soybean, correlations between canopy-level and average leaf phenotypes were generally lower. In rice, these correlations were weaker, although leaf angle exhibited increased association with canopy traits. Across all species, canopy-level traits were strongly intercorrelated, as were the remaining leaf traits, while leaf angle displayed a distinct pattern.

PVR correlation matrices were analyzed for each species (Fig. 10B). In maize, strong correlations were observed between leaf count and plant height, canopy width and leaf length, and leaf length and width. In soybean, leaf length, leaf width, and canopy width were highly correlated. In rice, strong correlations appeared between plant height and canopy width, leaf length and width, and leaf count and leaf angle.

## 4 Discussions

### 4.1 Unveiling crop growth dynamics and species variations through time-series phenotyping

This study examines how time-series phenotyping reveals both similarities and differences across species. Maize and rice show similar plant height and canopy width, generally greater than soybean (Fig. 5A), consistent with previous research suggesting that maize, as a tall crop, often develops greater height and canopy expansion compared to other species [39]. In contrast, soybean's branched architecture and smaller stature result in higher leaf count, reflecting the interplay between branching and internode elongation [40]. Maize shows a “plateau phase” in leaf count, with rapid early leaf initiation followed by slower growth. For leaf angle (Fig. 5B), soybean has the largest average angle, likely an adaptation to dense planting [41], while fluctuating angles in maize and rice suggest stronger environmental influences such as light and temperature.

Time-series phenotypic analysis based on PVR can capture dynamic crop growth. Across DAP, negative PVR values (Fig. 6A–C) may reflect environmental stresses such as nitrogen deficiency, water scarcity, or high temperatures, leading to temporary growth stagnation or regression [42], or could result from varietal differences or measurement errors. In maize, similar variation rates of average leaf length and width, especially on DAP 10, 14, and 19, suggest coordinated development, consistent with shared genetic and physiological regulation [43]. Analysis of individual leaves shows that mid-to-late-emerging leaves have higher variation rates than early-emerging leaves (Fig. S9), highlighting age-dependent growth variability [44]. Comparison across maize, soybean, and rice (Fig. S10) reveals species-specific strategies: maize's high canopy width variation indicates rapid canopy expansion; rice's high leaf count variation reflects tillering-driven growth; and soybean's high variation in plant height, leaf length, and width reflects its complex growth dynamics shaped by branching, internode elongation, and leaf morphology. Overall, PVR fluctuations reflect integrated responses to internal and external conditions.

The interpretation of these time-series patterns is shaped by methodological choices and parameter settings at each processing step. Parameters for skeleton extraction and pruning influence topological stability and organ delineation, which remain robust for sparse seedling canopies but may be challenged in denser architectures. The centroid-based organ matching strategy presumes gradual structural change, rendering it most suitable for short-term observations. PVR emphasizes relative temporal variation rather than absolute trait magnitude, making it sensitive to the observation interval and measurement noise. Despite these limitations, the consistent trends observed across species indicate that the proposed framework offers a reliable foundation for comparative time-series phenotyping during early growth stages. Given the sparse canopies of the seedlings analyzed here, centroid-based matching is adequate, although more sophisticated approaches incorporating organ shape, orientation, or topological constraints could be applied in denser or more complex canopies. This choice provides a balance between methodological simplicity and computational efficiency, aligning with the structural characteristics of early-stage crop growth.

### 4.2 Leaf-level photosynthetic contribution analysis provides insights into crop growth patterns and photosynthetic strategies

This study analyzes leaf-level time-series photosynthetic contributions in maize, soybean, and rice, revealing species-specific growth patterns and photosynthetic strategies. In maize (W64A), the first 2-3 leaves contribute most to photosynthesis, consistent with the concept of “functional leaves” [45]. Positioned at the top of the canopy, these leaves receive optimal light due to apical meristem location, entering the photosynthetically active radiation zone as the plant grows, in line with the Beer-Lambert Law [46,47]. Newly emerged upper leaves thus achieve higher photosynthetic rates [48], which peak before declining due to shading or senescence [49]. Non-zero contribution rates provide insights into organ developmental dynamics [50,51], and the rising-then-falling light-use efficiency reflects dynamic photosynthesis during leaf development [52,53]. In soybean (D21116), time-series contributions are intermediate between maize and rice. Early-stage leaves 3 and 4 dominate photosynthesis before DAP 12, reflecting soybean's branching pattern where new leaves are not always apical [54]. As growth continues, upper leaves assume larger contributions, resembling maize [55], indicating a flexible response to heterogeneous light environments [56]. In rice (M107), leaf 1 and leaf 3 maintain consistently high contributions, contrasting maize's dynamic shifts. New leaves emerge from both the main stem and tillers, while functional leaves such as the flag leaf and second- and third-to-last leaves contribute significantly to photosynthesis [57]. Positioned lower in the canopy, these leaves promote a balanced distribution of photosynthetic activity across layers, reflecting rice's distinct C3 growth strategy and reduced apical dominance (Fig. 9).

The differences in time-series photosynthetic contributions among maize, soybean, and rice reflect species-specific photosynthetic traits or responses [58]. In maize, photosynthesis is concentrated in the upper canopy, corresponding to high-light environments. Rice shows stable contributions from specific leaves throughout its life cycle, likely linked to growth under low-to medium-light conditions. Soybean exhibits intermediate, variable contributions reflecting canopy light distribution. Overall, maize displays more dynamic photosynthetic patterns under high light, while rice and soybean maintain relatively stable performance, with soybean showing greater variability. These observations align with previous modeling studies, such as MaluSim [59], which simulates canopy photosynthesis integrating physiological and environmental factors, and LiDAR-based canopy modeling that captures spatial light heterogeneity [60]. Applying similar modeling approaches could enhance interpretation of time-series data and strengthen quantitative links between canopy architecture and photosynthetic performance.

It should also be noted that this study involves both C3 and C4 species, which differ in photosynthetic pathways and responses to light, temperature, and CO_2_. Therefore, the observed interspecific differences primarily reflect species-specific photosynthetic behaviors rather than direct adaptive responses. Future work combining physiological modeling and controlled experiments will be essential to clarify the mechanisms behind these time-series dynamics. All point clouds were collected during the early seedling stage, representing short-term structural and functional dynamics. Incorporating multi-stage temporal data in future studies will enable modeling of growth rhythms and key phenological events over the full life cycle.

## 5 Conclusion

This study proposes a framework integrating Laplacian skeleton-based organ-level 3D segmentation, time-series leaf matching, phenotypic variation rate (PVR) calculation, and leaf-level photosynthetic assessment. This approach may enable tracking dynamic phenotypic changes and estimating organ-specific photosynthetic contributions, providing preliminary insights into crop growth strategies and light adaptation. Observations suggest species-specific patterns: in maize, upper leaves tend to contribute more to photosynthesis; in rice, certain leaves maintain relatively stable contributions; and in soybean, contributions are intermediate and variable, potentially reflecting branching architecture and heterogeneous light capture. PVR appears useful for quantifying short-term growth dynamics and highlighting differences among species. However, these findings are based on early seedling-stage data, and organ matching relies on centroid distances, which may be less robust in dense or complex canopies. Future work should expand datasets across growth stages and species, improve organ-level feature extraction, and combine physiological modeling with controlled experiments to better interpret the mechanisms underlying phenotypic and photosynthetic variation. Overall, this framework offers a cautious yet promising approach for exploring dynamic crop growth and photosynthetic strategies, with potential applications in precision agriculture and crop improvement.

## Author contributions

J.Z. and Y.Z. wrote the manuscript with input from all the authors. J.Z., Y.Z., M.Z., Q.S. and M.Z. performedthe experiments and phenotyping under X-G.Z. and M.W.’s super-vision. J.Z., Y.Z. and M.Z. performed the data analysis and modeling. This work represents a collaborative effort between two research groups, both co-first authors contributed equally to this work, and both co-corresponding authors contributed significantly to the paper's revision, and their complementary roles were essential to the success of this study.

## Funding

This work was supported by National Natural Science Foundation of China (No. U22A20464), Strategic priority research program of the Chinese Academy of Sciences (No. XDB0630000) and the 2115 Talent Development Program of China Agricultural University.

## Declaration of competing interest

The authors declare that they have no known competing financial interests or personal relationships that could have appeared to influence the work reported in this paper.

## Data Availability

A subset of the code and dataset used in this study is publicly available on our GitHub repository: https://github.com/JiarenZhou/LTPCDCCM. The complete time-series 3D crop point cloud dataset can be downloaded from https://pan.baidu.com/s/1mNSDz4F0ZjOwmqzMuXozSQ?pwd&equals;1234.
