# Supplementary material for: Leveraging time-series point clouds for dynamic crop canopy monitoring: Quantifying phenotypic variability and assessing leaf-level photosynthetic contributions
Source: Plant Phenomics. 2026 Mar 4;8(2):100194. doi: 10.1016/j.plaphe.2026.100194 (PMC13316240; doi:10.1016/j.plaphe.2026.100194)
Supplement: Multimedia component 1 [file mmc1.pdf]

Supplementary materials

Additional Supporting Information may be found online in the Supporting Information section at the end of the article.

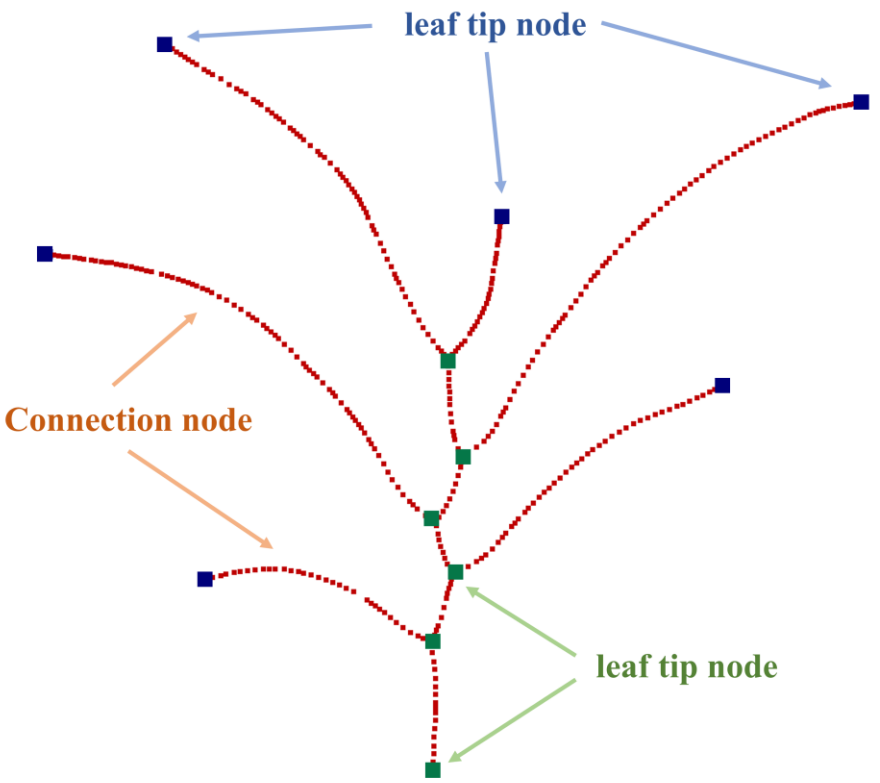

Fig.S1 Schematic diagram of stem skeleton key point extraction, the corresponding relationship between node colors is as follows: stem nodes-green, leaf tip nodes-blue, connection nodes-red.

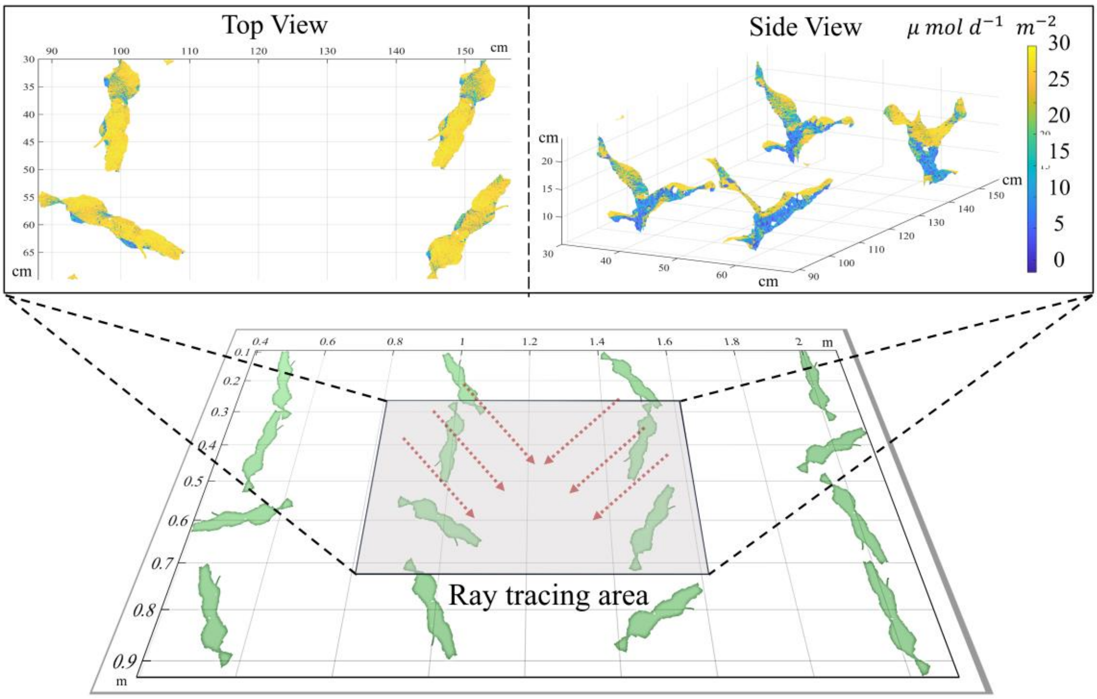

Fig.S2 Schematic representation of the virtual crop canopy population model, with the gray area indicating the range of ray tracing. By applying the ray tracing algorithm, the light distribution across the entire canopy space can be accurately captured, enabling the calculation of photosynthetic rates in different regions. The figure provides a visualization of the spatial distribution of canopy photosynthetic rates from both a top-down perspective and a 3D view.

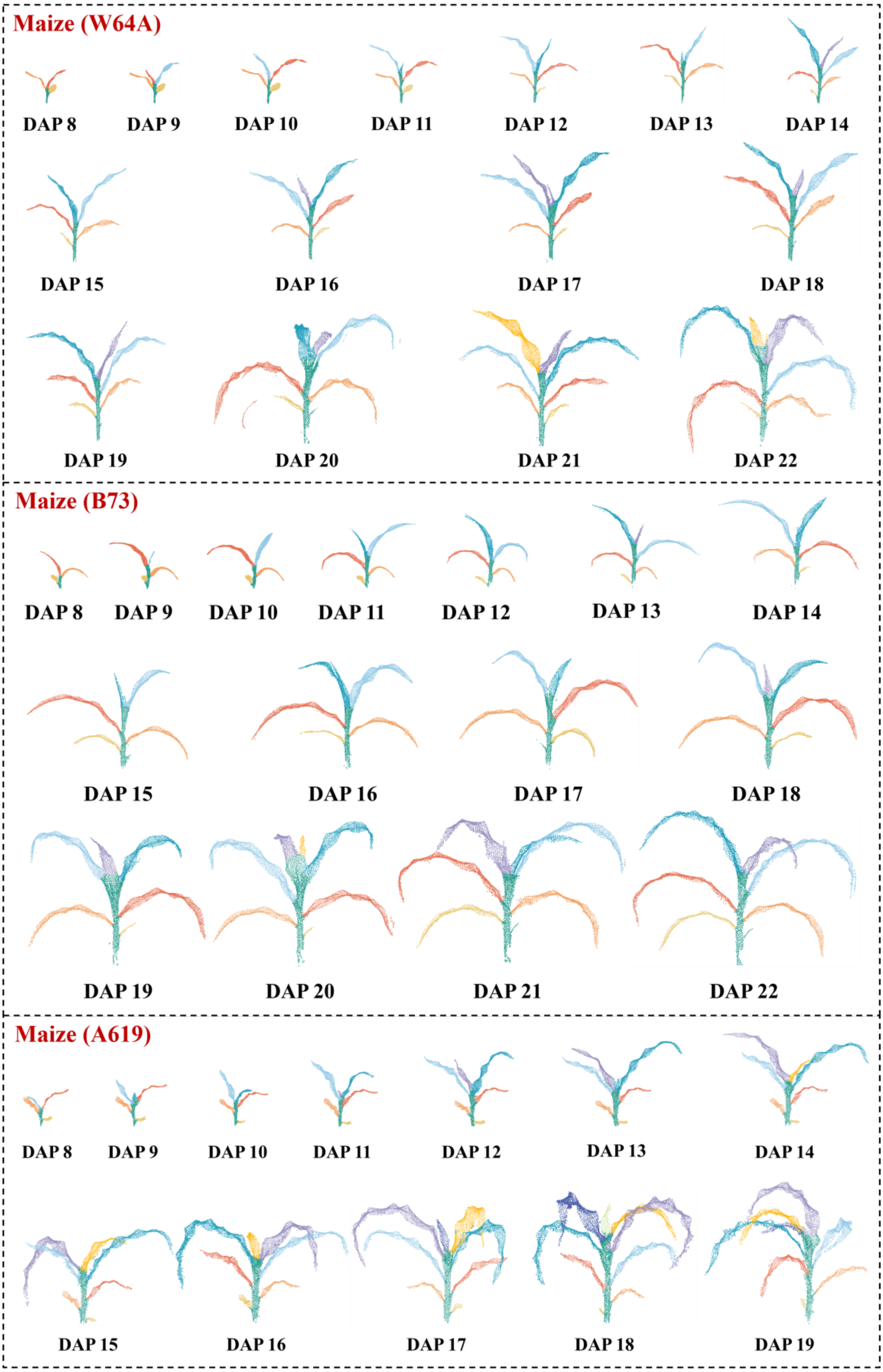

Fig.S3 Segmentation effect of self-collected maize dataset, varieties include W64A, B73 and A619.

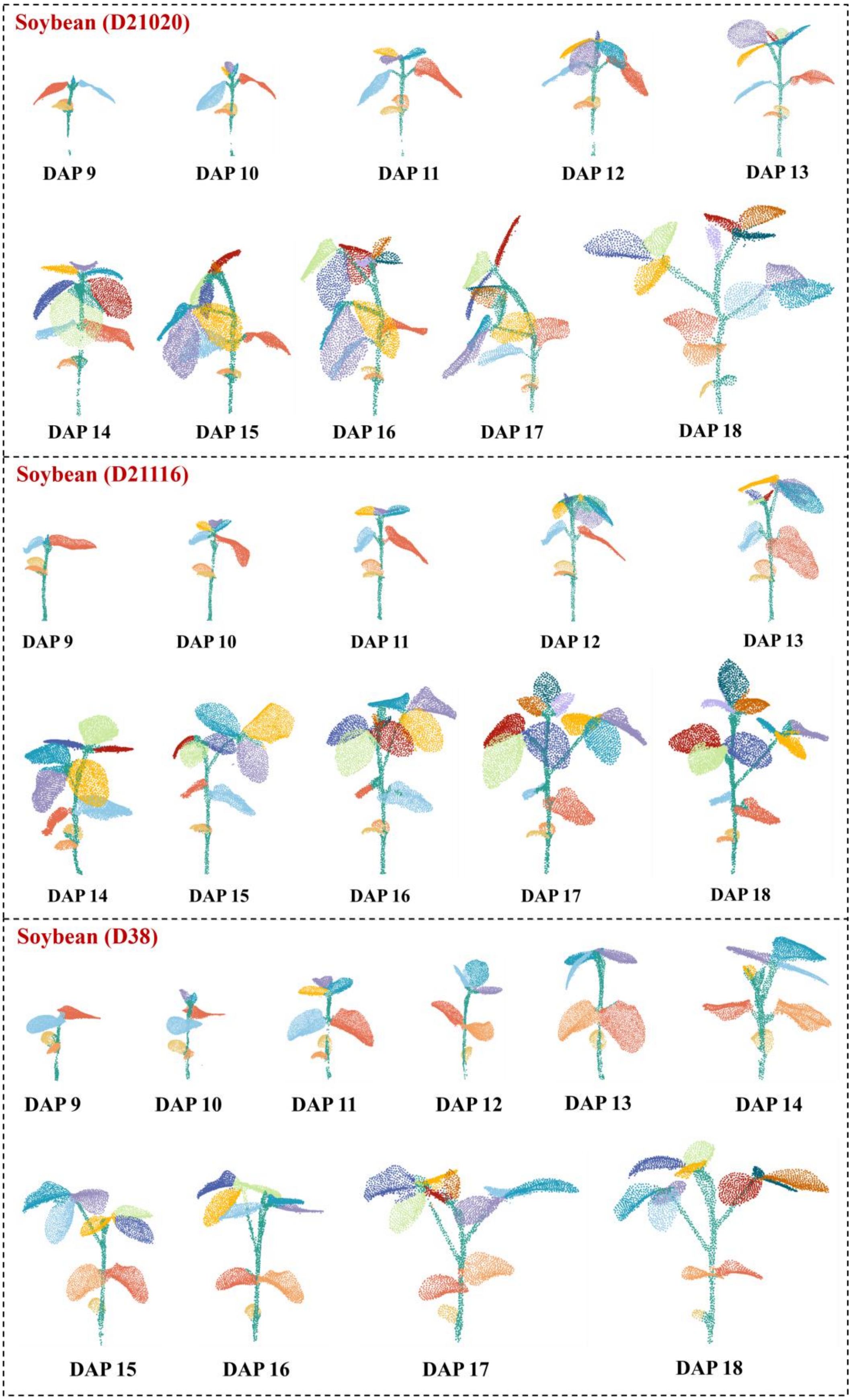

Fig.S4 Segmentation effect of self-collected soybean dataset, varieties include D21020, D21116 and D38.

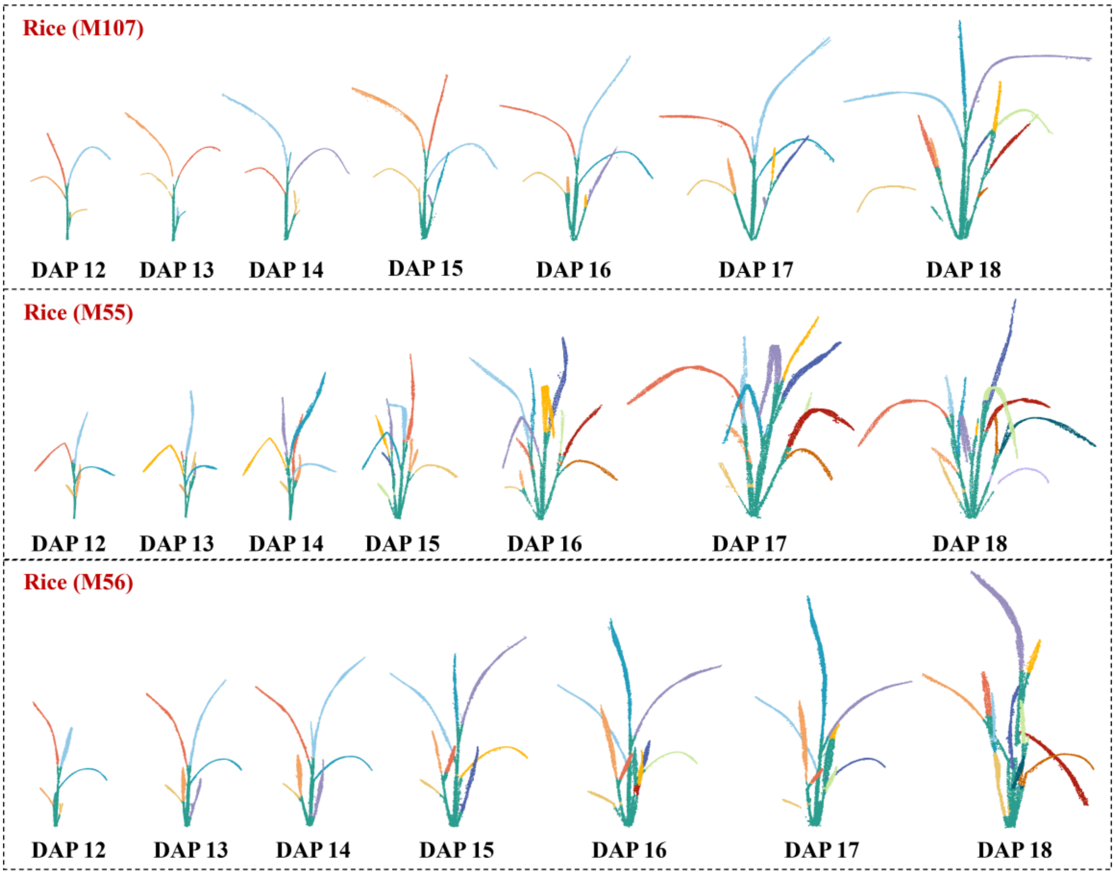

Fig.S5 Segmentation effect of self-collected rice dataset, varieties include M107, M55 and M56.

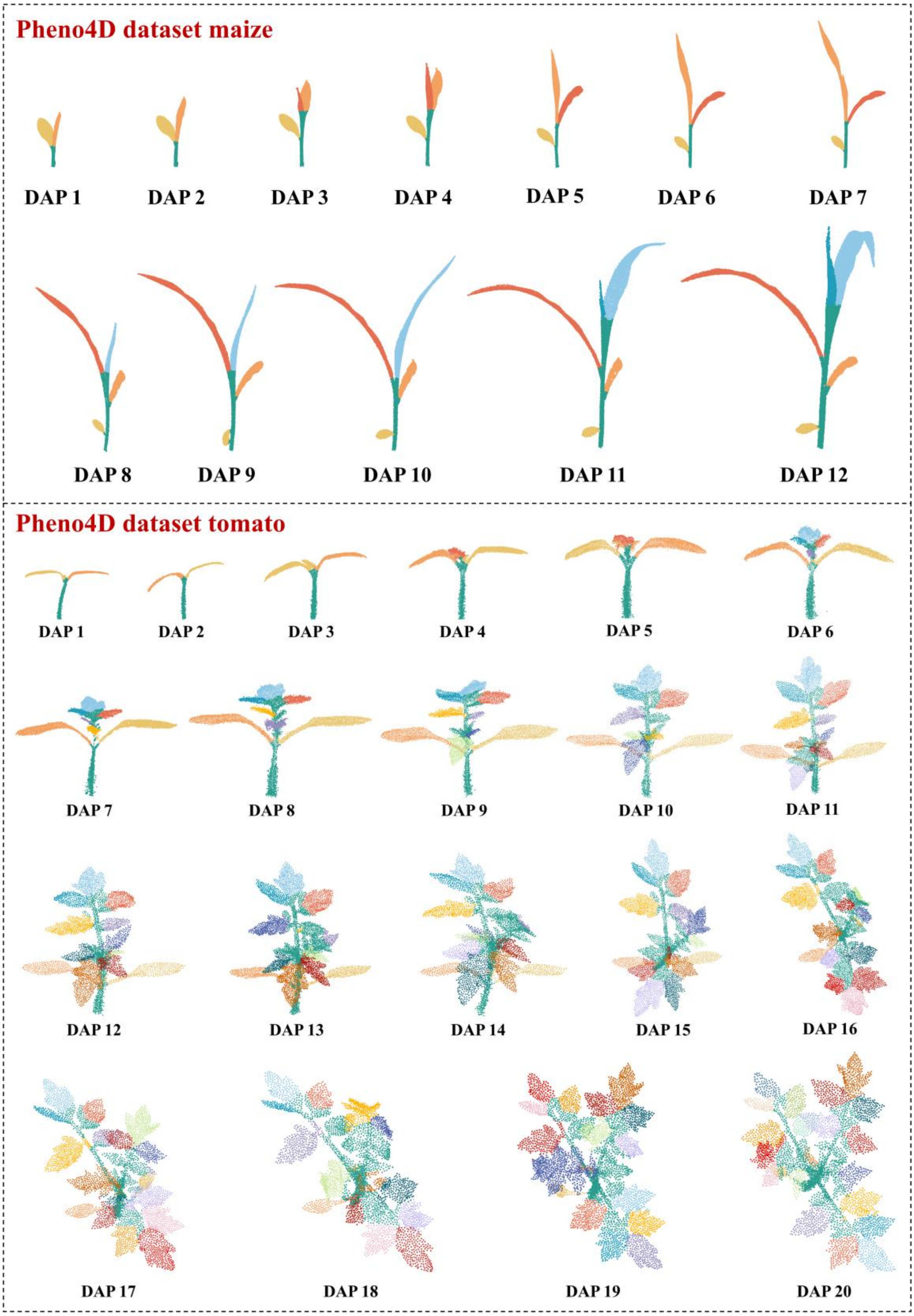

Fig.S6 Segmentation effect of public dataset Pheno4D, varieties include maize and tomato.

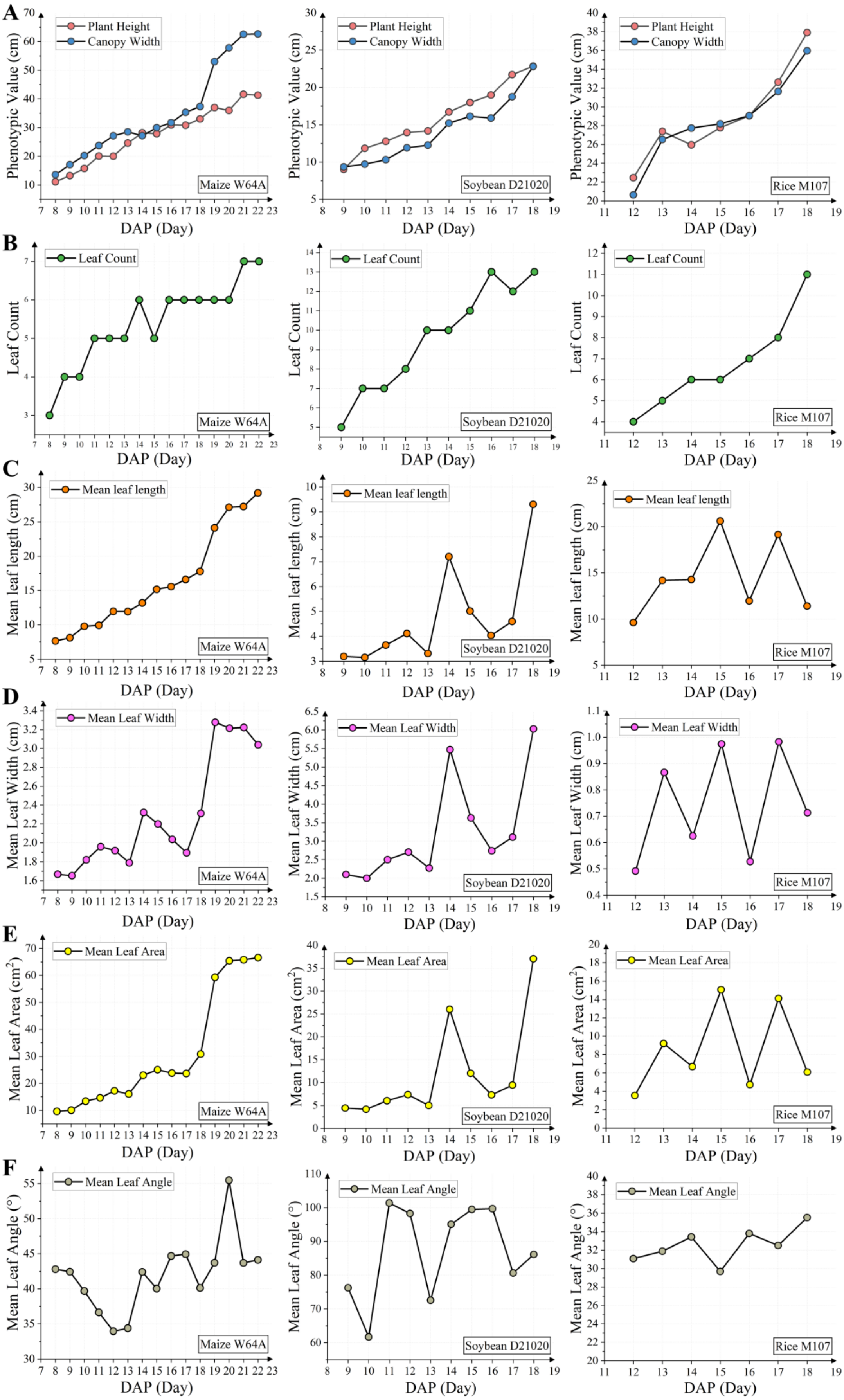
A
Plant Height
Canopy Width
Phenotypic Value (cm)
Maize W64A
Soybean D21020
Rice M107
DAP (Day)
B
Leaf Count
C
Mean leaf length
Mean leaf length (cm)
D
Mean Leaf Width
Mean Leaf Width (cm)
E
Mean Leaf Area
Mean Leaf Area (cm²)
F
Mean Leaf Angle
Mean Leaf Angle (°)

Fig.S7 Time-series phenotypic changes of the three crops. (A) Plant height. (B) Canopy width. (C) Leaf count. (D) Average leaf length. (E) Average leaf width. (F) Average leaf angle.

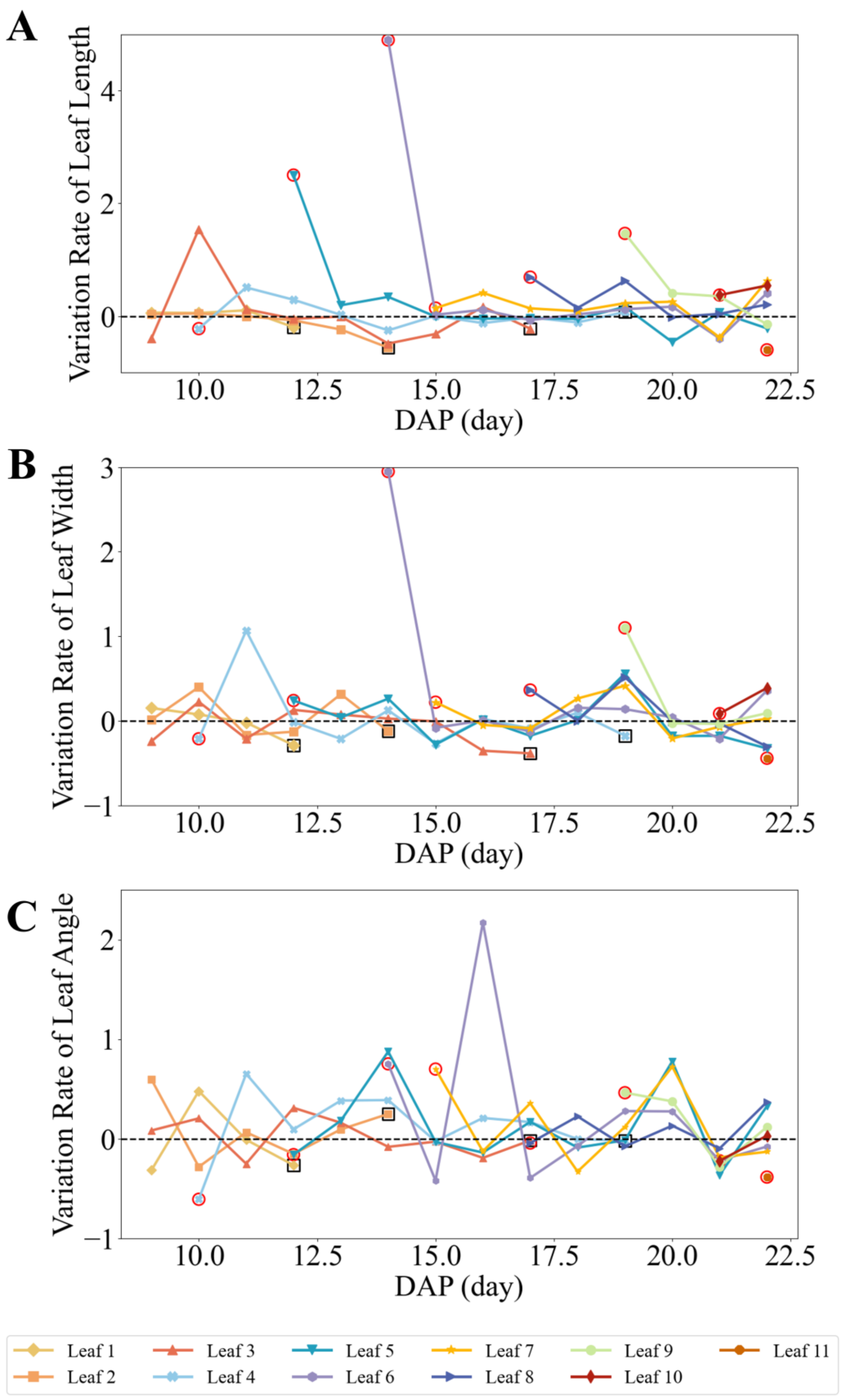

Fig.S8 Time-series phenotypic variation rates of each maize leaf. (A) Variation rate of leaf length. (B) Variation rate of leaf width. (C) Variation rate of leaf angle.

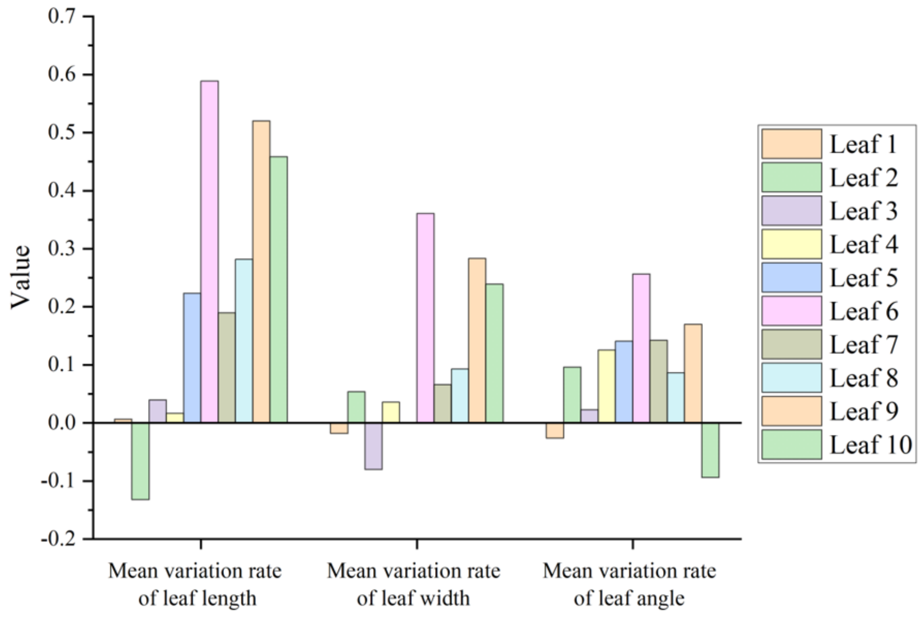

Fig.S9 Comparison of the mean variation rates of leaf length, leaf width, and leaf angle among maize leaf instances.

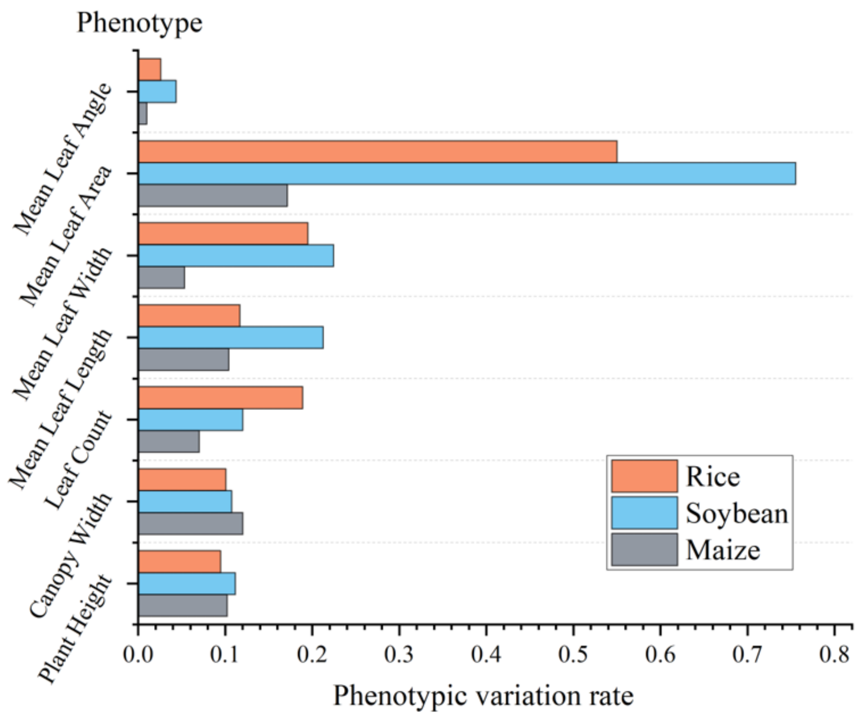

Fig.S10 Comparison of the average phenotypic variation rates among three species.

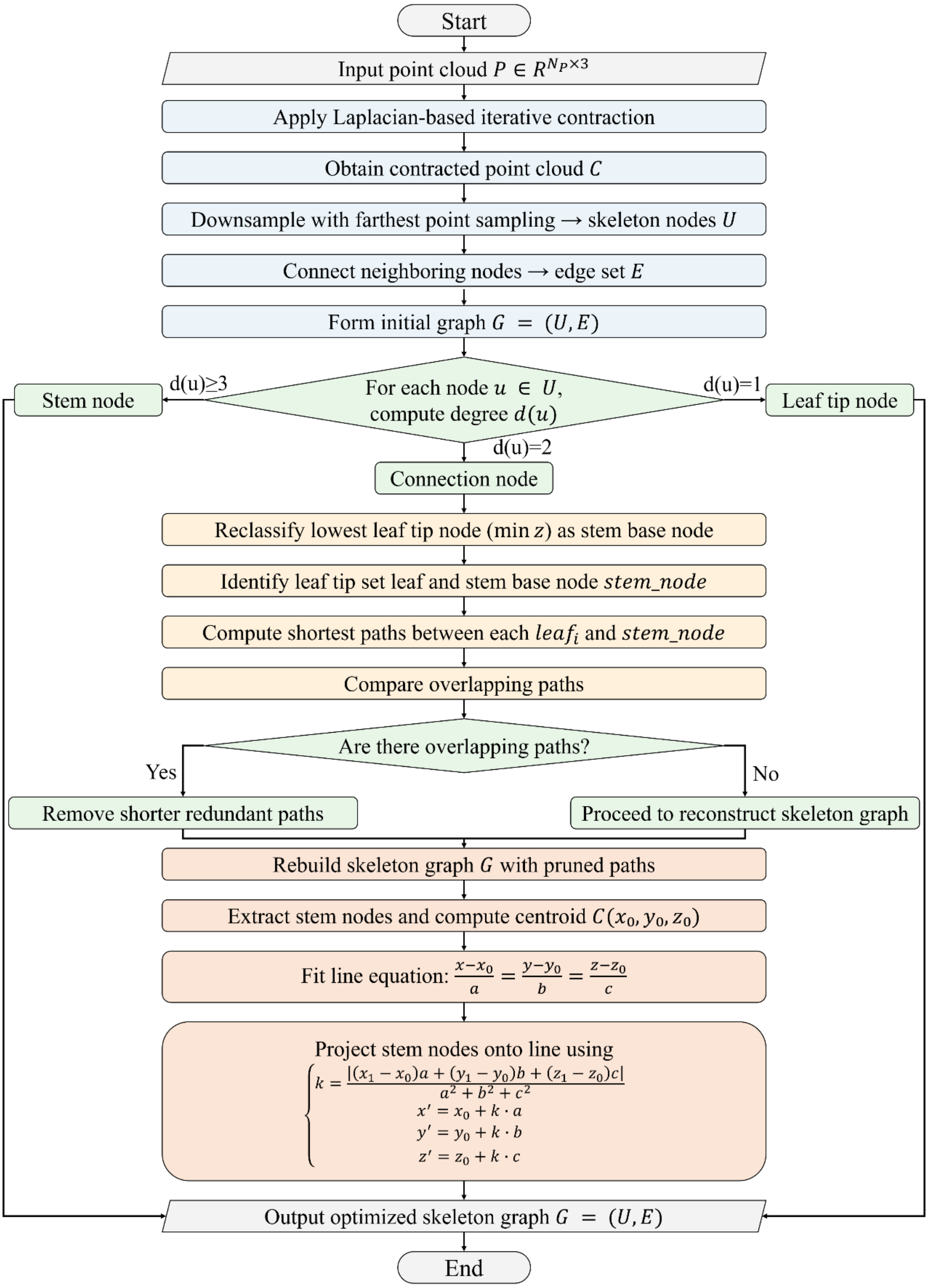

Fig.S11 Workflow of the crop skeleton extraction and optimization algorithm.

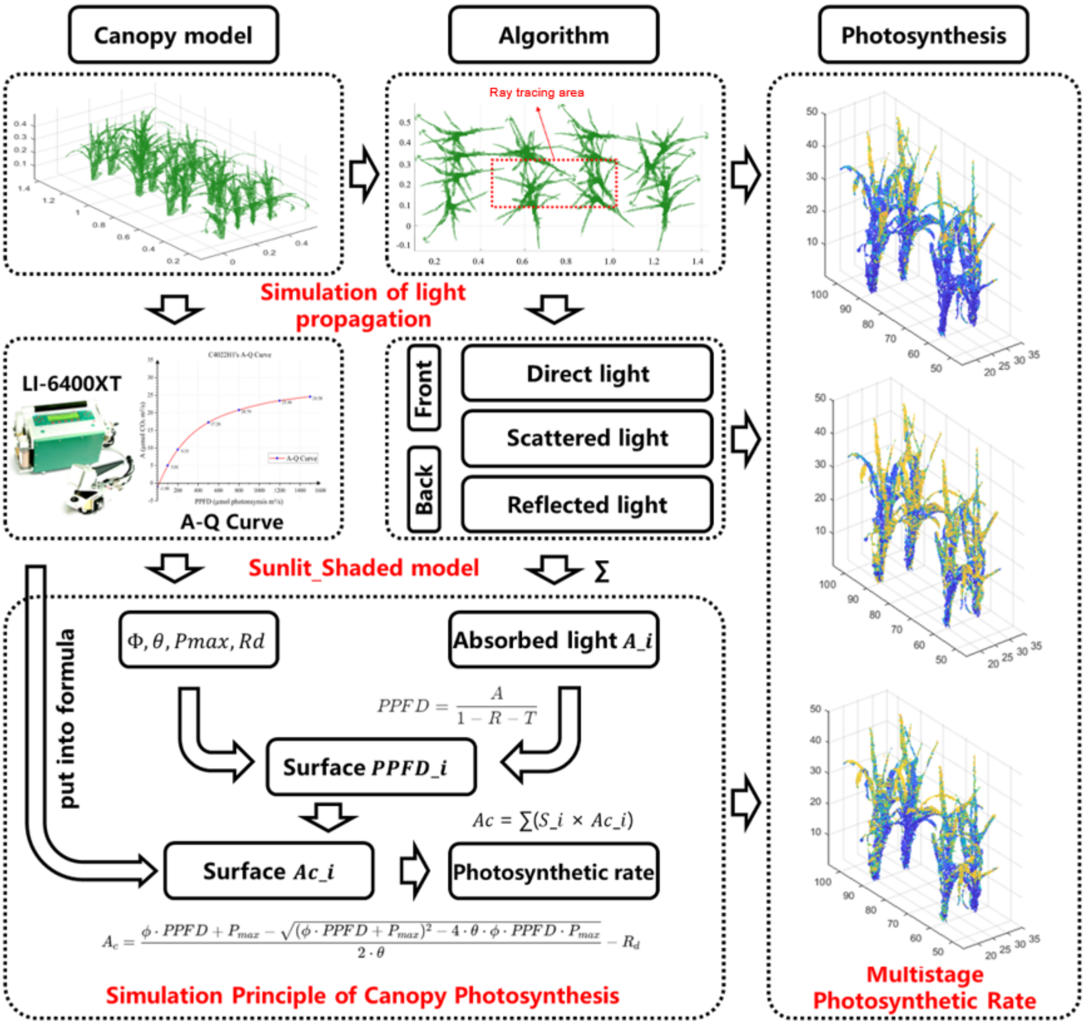

Fig.S12 The principle of using the FastTracer software for photosynthetic simulation
